# Large-Area, High-Specific-Power Schottky-Junction Photovoltaics from CVD-Grown Monolayer MoS_2_

**DOI:** 10.1021/acsami.2c01650

**Published:** 2022-05-20

**Authors:** Kazi M. Islam, Timothy Ismael, Claire Luthy, Orhan Kizilkaya, Matthew D. Escarra

**Affiliations:** † Department of Physics and Engineering Physics, Tulane University, New Orleans, Louisiana 70118, United States; ‡ Center for Advanced Microstructures & Devices, Louisiana State University, Baton Rouge, Louisiana 70806, United States

**Keywords:** 2D materials, transition metal dichalcogenides, photovoltaics, Schottky junction, specific power, chemical vapor deposition

## Abstract

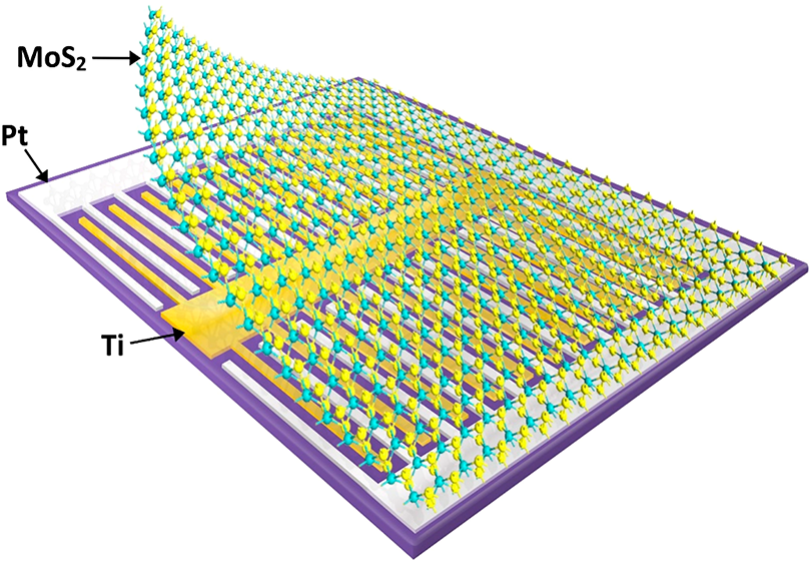

The deployment of two-dimensional (2D) materials for solar energy conversion requires scalable large-area devices. Here, we present the design, modeling, fabrication, and characterization of monolayer MoS_2_-based lateral Schottky-junction photovoltaic (PV) devices grown by using chemical vapor deposition (CVD). The device design consists of asymmetric Ti and Pt metal contacts with a work function offset to enable charge separation. These early stage devices show repeatable performance under 1 sun illumination, with *V*_OC_ of 160 mV, *J*_SC_ of 0.01 mA/cm^2^, power conversion efficiency of 0.0005%, and specific power of 1.58 kW/kg. An optoelectronic model for this device is developed and validated with experimental results. This model is used to understand loss mechanisms and project optimized device designs. The model predicts that a 2D PV device with ∼70 kW/kg of specific power can be achieved with minimum optimization to the current devices. By increasing the thickness of the absorber layer, we can achieve even higher performance devices. Finally, a 25 mm^2^ area solar cell made with a 0.65 nm thick MoS_2_ monolayer is demonstrated, showing *V*_OC_ of 210 mV under 1 sun illumination. This is the first demonstration of a large-area PV device made with CVD-grown scalable 2D materials.

## 1 Introduction

The strong light–matter interaction in two-dimensional (2D) transition metal dichalcogenides (TMDCs) such as MoS_2_ results in very high absorptance and photogeneration in these materials, making them suitable for flexible and ultralight photovoltaics (PV) and other optoelectronic devices.^1,2^ In this paper, we present a Schottky-junction PV device using large-area 2D MoS_2_ with lateral (in-plane) current flow and asymmetric contacts.^3,4^ Bernardi et al. predict that subnanometer thick 2D TMDC materials can achieve 1 order of magnitude higher sunlight absorption per unit thickness than GaAs and thus can achieve 1–3 orders of magnitude higher power densities than the best existing ultrathin solar cells.^5^ These properties show the potential for 2D PV for use in weight and volume constrained applications, such as in space-based, building-integrated, and vehicle-integrated solar energy conversion applications.

Jariwala et al. predict that monolayer TMDC-based PV cells can achieve a maximum of 27% power conversion efficiency using detailed balance theory.^6^ However, to date, the power conversion efficiency in all-2D material PV devices has been in the ∼1–5% range, achieved with hundreds of nanometers thick layers that are more like thin films than 2D layers.^7^ Open-circuit voltage (*V*_OC_) is a critical parameter for PV that gives insight into device quality and performance potential. Fontana et al. demonstrated Schottky-junction PV with exfoliated ∼50 nm thick MoS_2_ and Pd and Au contacts and achieved a *V*_OC_ of 100 mV.^8^ Choi et al. demonstrated a lateral Schottky-junction device with Pd and Cr/Au contacts and exfoliated multilayer MoS_2_.^9^ Their device as-is showed a *V*_OC_ of 80 mV; however, with AuCl_3_ doping, they were able to improve the *V*_OC_ to 200 mV. Wi et al. also utilized multilayer exfoliated MoS_2_ to demonstrate a plasma-induced p-doping in a p–n junction PV device with a *V*_OC_ of 130 mV and power conversion efficiency of 0.34% as-is and was treated with CHF_3_ resulting in improvement of these values to 280 mV and 2.8%, respectively.^10^ Cora et al. demonstrated a vertical Schottky-junction PV device with exfoliated 16 nm WS_2_ and asymmetric Au and Ag contacts to achieve 256 mV of *V*_OC_ and a power conversion efficiency of 0.46% under AM1.5G solar spectrum.^11^ To date, all demonstrations of PV utilizing van der Waals stacked TMDCs have utilized small (∼0.0025 mm^2^ in the largest reported case) exfoliated flakes.^7,11,12^ In this work, we demonstrate a lateral Schottky-junction PV device with chemical vapor deposition (CVD)-grown monolayer MoS_2_ and Ti and Pt contacts. This is the first work to report 2D PV device performance using CVD-grown large-area, scalable 2D materials—a prerequisite for practical deployments of 2D PV for solar energy conversion.

## 2 Experimental and Computational Methods

### 2.1 Device Structure

**Figure 1:**
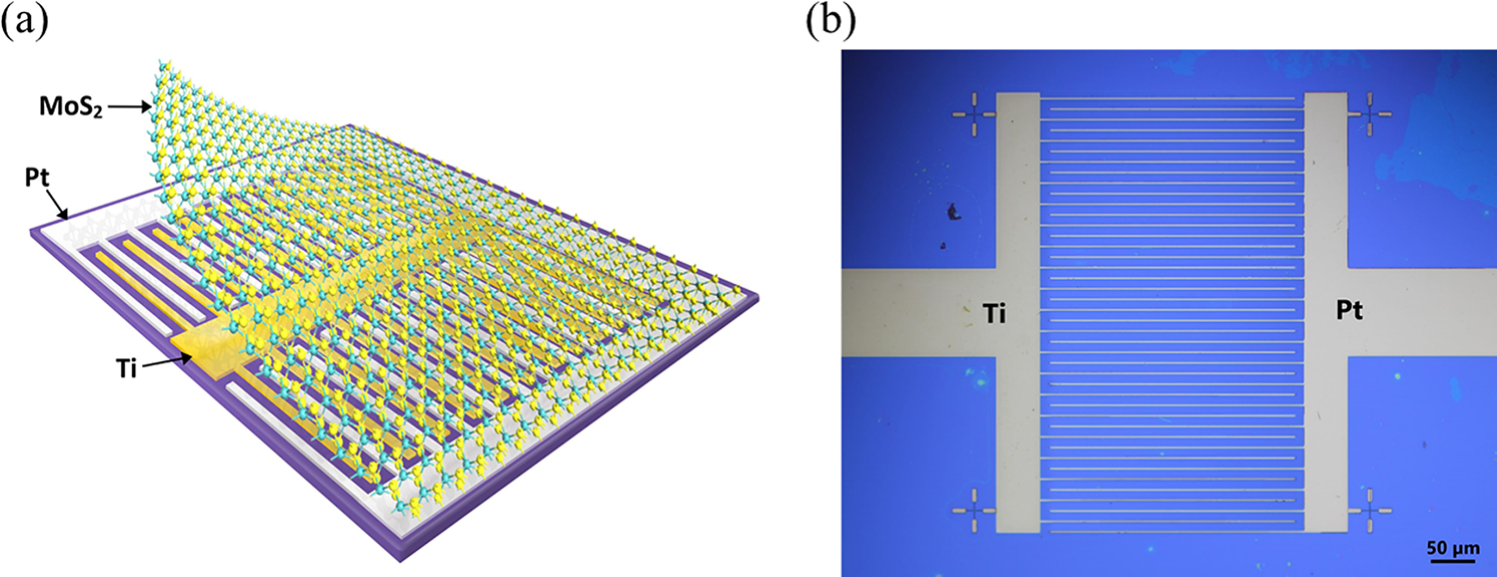
(a) Schematic of a Schottky-junction PV device with asymmetric contacts and monolayer MoS_2_. (b) Fabricated asymmetric contact Schottky-junction solar cells with Ti and Pt contacts. In the depicted device, the fingers span over a 300 μm × 500 μm active cell area and are each 2 μm wide with a 10 μm channel between the fingers.

The Schottky-junction PV devices demonstrated in this work use asymmetric contacts for carrier (electron and hole) separation under illumination, without requiring a p–n junction. Figure 1 shows a schematic of a device with asymmetric Ti and Pt contacts and monolayer MoS_2_. The differences in work function between the metals is useful for forming opposing Schottky barriers at the contacts, achieving carrier separation with no applied source–drain bias. A SiO_2_-on-Si substrate is patterned by using electron beam lithography, followed by the deposition of the first metal and a liftoff step. The set of interlocking fingers is produced by precise alignment of a second electron beam lithography pattern and a second metal deposition and liftoff. Monolayer MoS_2_ is grown on a sapphire substrate by using tube furnace CVD with MoO_3_ and S_2_ powder precursors, exhibiting precise monolayer thickness control and uniformity over large area (>1 cm^2^). As-grown films are characterized for physical and optoelectronic properties, such as thickness via atomic force microscopy (AFM), photoluminescence, Raman spectroscopy, and carrier mobility as shown in the Supporting Information, section S1. Details on the film growth and characterization are published in our previous report.^13^ The measured mobility of the films used in this work is 1–3 cm^2^ V^-1^ s^-1^, as also shown in section S1. To complete the fabrication of these 2D PV devices, these MoS_2_ monolayers are transferred^14^ onto the metal contacts as illustrated in Figure 1a.

Figure 1b shows the fabricated asymmetric contact Schottky-junction solar cells with Ti (50 nm thick) and Pt (50 nm thick) contacts. The interlocking fingers in this device are 2 μm wide with 10 μm channel between them, but devices with variable channel lengths are fabricated as shown later on in this paper. Unless otherwise stated, the performance of 1 μm channel length devices is presented and analyzed throughout the paper. Devices were fabricated in arrays to generate more measurable devices for the same MoS_2_ film, enabling measurements with statistical significance. Further details on the fabrication process are described in the section S2. The device architecture is inspired by an all-in-one optoelectronic device concept, the details of which are shown in section S3.

Schottky-junction PVs are fundamentally different from traditional p–n junction solar cells in terms of how their built-in voltage is formed.^3^ In a conventional solar cell, a p-type and a n-type semiconductor materials are brought together to form a p–n junction. Because of the offsets in the p-type and n-type materials’ Fermi levels, a built-in potential difference is created, which results in electron–hole pair carrier separation. In a Schottky-junction solar cell, however, the built-in voltage is formed by the offset between the Fermi level of the semiconductor and the work functions of the metal contacts. At the interface between the semiconductor and metal, band bending happens due to this offset, and a so-called Schottky barrier is formed.

**Figure 2:**
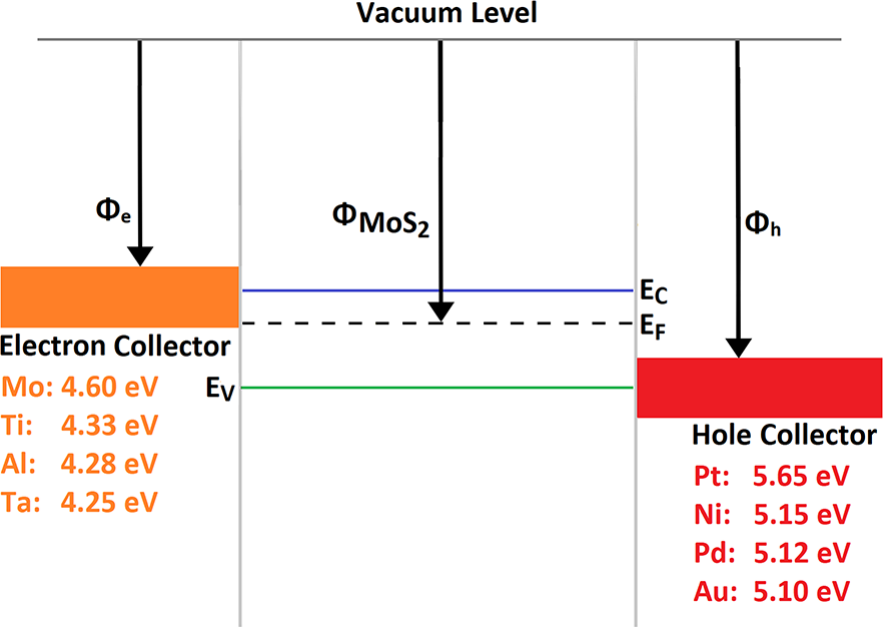
Band structure schematic for monolayer MoS_2_, showing metal work function requirements for carrier separation, with the range of electron-selective work functions in orange and hole-selective work functions in red. Possible low and high work function metals are listed on the left and right, respectively.

Two different metal contact materials need to be carefully chosen to drive carrier separation in a Schottky-junction PV device, such that each metal aligns to either the conduction or valence band of MoS_2_ for electron or hole collection, respectively. For this work, metals with various work functions are studied, as shown in Figure 2. Several low work function metals, such as Mo, Ti, Al, Sc, and Yt, are considered for electron collection (denoted as Φ_e_ in the rest of the paper), while high work function metals such as Co, Ni, Au, Pd, and Pt are considered for hole collection^15^ (denoted as Φ_h_ in the rest of the paper). In the middle of Figure 2 is shown the band structure of monolayer MoS_2_, having a bandgap of 1.85 eV. The possible metal candidates for effective carrier separation and their work functions are shown on the right and left.^16^

### 2.2 Optoelectronic Device Model

A 1D finite element analysis device model is built by using the COMSOL Multiphysics simulation tool’s Semiconductor Module. This model simulates a MoS_2_-based solar cell device with asymmetric Ti and Pt contacts. The model uses homogeneous doping in the active MoS_2_ material via the Analytical Doping Model built in to COMSOL. Equation 1 is used to calculate photogeneration that includes the AM1.5D solar irradiance and MoS_2_ absorption spectra. The absorption spectra are calculated in Equation 2 by using the extinction coefficient of monolayer MoS_2_. The complex refractive index for monolayer MoS_2_ is measured and published in our previous report.^13^Equation 3 shows the photon flux that is used to calculate the photogeneration.
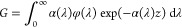
1
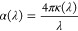
2
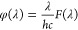
3Here, *z* is the depth into the device while the lateral junction is formed in the *x*–*y* plane between the contacts; given the monolayer thickness of the device, the generation profile is constant in *z*. λ is the wavelength in vacuum, κ(λ) is the wavelength-dependent extinction coefficient, and *F*(λ) denotes the AM1.5D spectral irradiance. With 100% IQE, i.e., all the electron–hole pairs that are generated are collected, a maximum of 1.34 mA/cm^2^*J*_SC_ is estimated for monolayer MoS_2_-based solar cells.^13^ To account for realistic collection losses, the Shockley–Read–Hall (SRH) recombination model is implemented with a trap-assisted recombination feature, also built into the COMSOL solver. As SRH recombination has been shown to be the dominant recombination mechanism for 2D materials-based photovoltaics,^12^ Auger recombination was not included in our model. The SRH model in this solver is parametrized with carrier (electron and hole) lifetimes. The model does not require separate definition of the density of traps, as the carrier lifetime in the model takes into account defect concentrations/density of traps (higher defect density results in lower lifetime) among other parameters.

**Figure 3:**
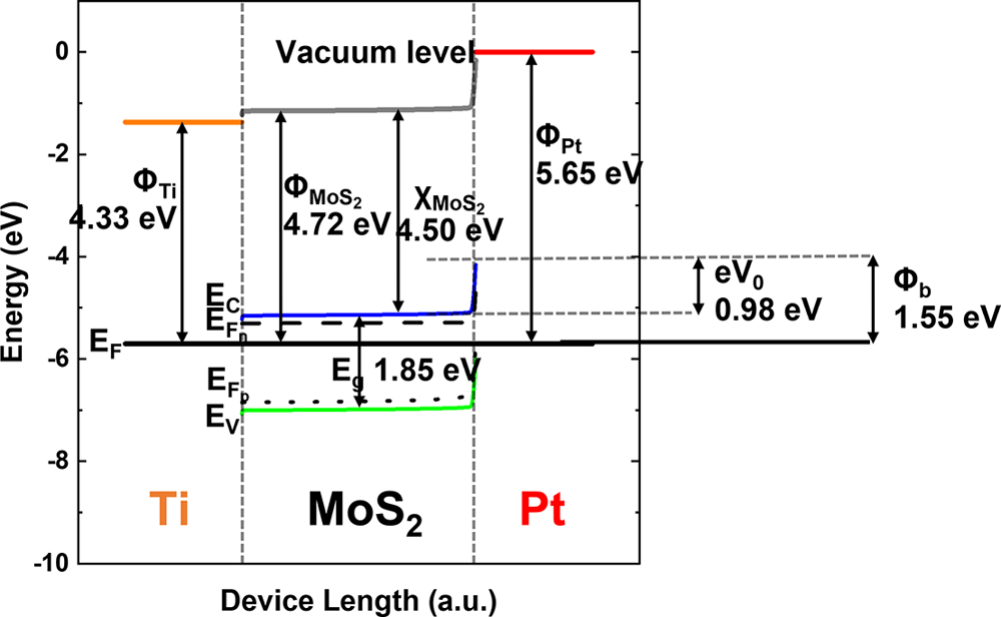
Band structure of a Ti–MoS_2_–Pt Schottky-junction solar cell showing the asymmetric band bending at the metal–semiconductor interfaces. The larger bend on the right side between MoS_2_ and Pt indicates a large Schottky barrier.

The model uses several nonparametrized inputs for the MoS_2_ layer, such as a thickness of 0.65 nm, doping concentration of 1 × 10^12^ cm^–2^, bandgap of 1.85 eV, electron affinity of 4.5 eV, relative permittivity of 3.5, and effective density of states 2.66 × 10^19^ cm^–3^ (electrons) and 2.86 × 10^19^ cm^–3^ (holes).^1,17,18^ Some other inputs into the model are parametrized and swept for realistic values that are obtained from either our experiments or literature, such as an electron mobility between 1 and 10 cm^2^ V^–1^ s^–1^ (extracted experimentally from a MoS_2_-based transistor by using the FET model) and a carrier lifetime of 1 μs, calculated from our experimentally measured diffusion length and mobility.^19^ The work functions of the asymmetric metal contacts are also parametrized, and their effect on the overall device performance is studied. The model then calculates the energy band structure diagram of the device, as shown in Figure 3. The band bending between MoS_2_ and the contact metals is demonstrated here. On the left side is the Ti contact with 4.33 eV work function that aligns well with the conduction band of MoS_2_; hence, an approximately ohmic contact forms at this metal–semiconductor junction. On the right side, Pt forms a large Schottky barrier with its 5.65 eV work function, as evident by the large band bending, which is essential for the carrier separation.

**Figure 4:**
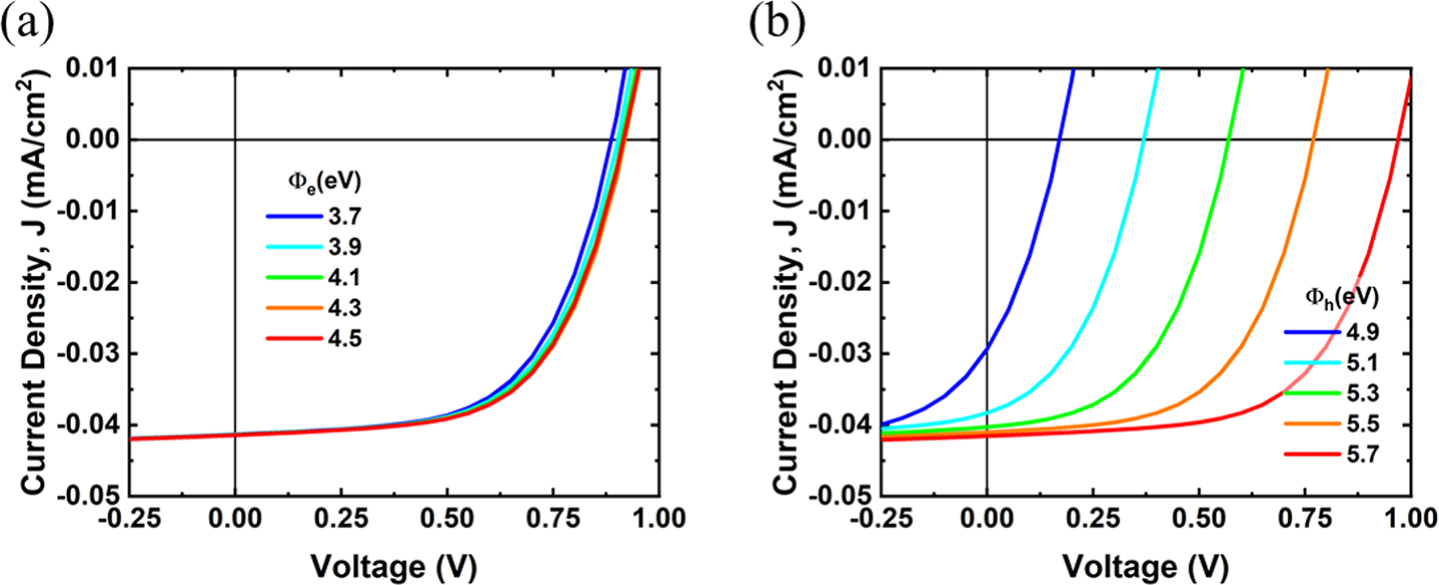
Simulated *J*–*V* plots of a Schottky-junction monolayer MoS_2_-based solar cell showing the effect of contact metal work functions for (a) the electron-selective contact (Φ_e_) and (b) the hole-selective contact (Φ_h_). For (a) the hole collector work function is set at 5.65 eV, and for (b) the electron collector work function is set at 4.33 eV.

Once the model is established, the current density–voltage (*J*–*V*) relationship of the device is studied under forward bias. Figure 4 shows the simulated *J*–*V* plots for the various work functions of the asymmetric contacts. As can be seen, the *V*_OC_ depends heavily on the work function of the hole-selective contact (Φ_h_) and not as much on the electron-selective contact (Φ_e_). Further modeling details and simulated results are shown in section S4.

## 3 Results and Discussion

### 3.1 Experimental Photovoltaic Performance

Ti and Pt are chosen as the contact metals because of their low (4.33 eV) and high (5.65 eV) work functions suitable for Schottky-junction formation, as discussed in the previous sections, along with their wide use to date for contacting 2D MoS_2_. These asymmetric contacts create the necessary band offsets at the metal–MoS_2_ interfaces between the Fermi levels of these metals and that of MoS_2_, thus driving the electrons toward Ti and holes toward Pt and separating the photogenerated carriers without any applied bias.

Devices were characterized for open-circuit voltage (*V*_OC_), short-circuit current (*I*_SC_), short-circuit current density (*J*_SC_), fill factor (FF), power conversion efficiency (η), series resistance (*R*_S_), shunt resistance (*R*_SH_), and specific power, all at room temperature. The devices were illuminated by monochromatic laser excitation with high concentration (shown in section S5) and the standard 1 sun AM1.5D spectrum in a solar simulator.

**Figure 5:**
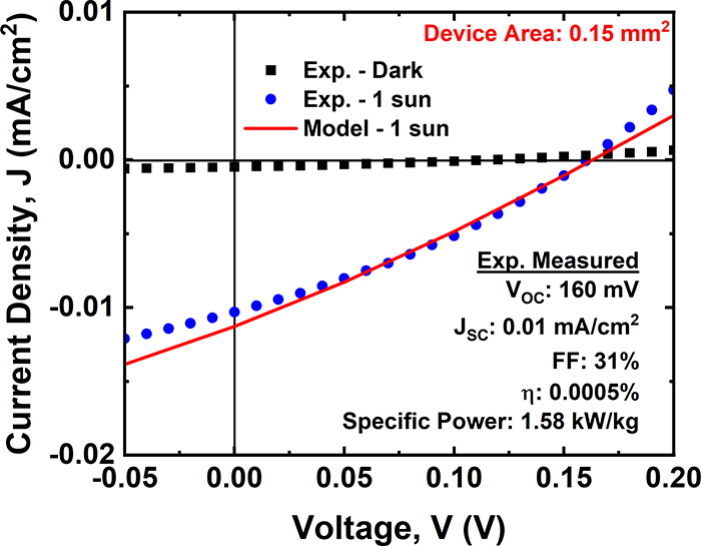
Performance of a Schottky-junction monolayer MoS_2_-based solar cell in the dark and under 1 sun equivalent AM1.5D illumination, measured at room temperature. The solar cell active area is 0.15 mm^2^ with 1 μm channels between asymmetric Ti and Pt grid fingers. The solid red line is a modeled *J*–*V* plot for the same solar cell structure.

Figure 5 shows the PV performance under standard 1 sun AM1.5D illumination conditions. As shown, the *V*_OC_ and *J*_SC_ are recorded as 160 mV and 0.01 mA/cm^2^, respectively. The fill factor is calculated as 31%, and the efficiency of this device is 0.0005%. The extracted series resistance is 8.9 × 10^3^ Ω·cm^2^, and the shunt resistance is 2.6 × 10^4^ Ω·cm^2^, calculated from the slope of the *J*–*V* curve at the open- and short-circuit conditions, respectively. The series and shunt resistance are both high relative to other 2D PV devices^7^ due to the lateral transport device architecture used in this work. This trade-off, achieving a desirable shunt resistance at the expense of a less desirable series resistance, was made to avoid pinhole shunting from defects in our CVD films. In addition to the large series resistance, several factors are limiting overall efficiency in these proof-of-concept devices, including relatively low photon absorption in a single monolayer device, hole collector work function reduction, and limited electronic transport due to material quality.

**Figure 6:**
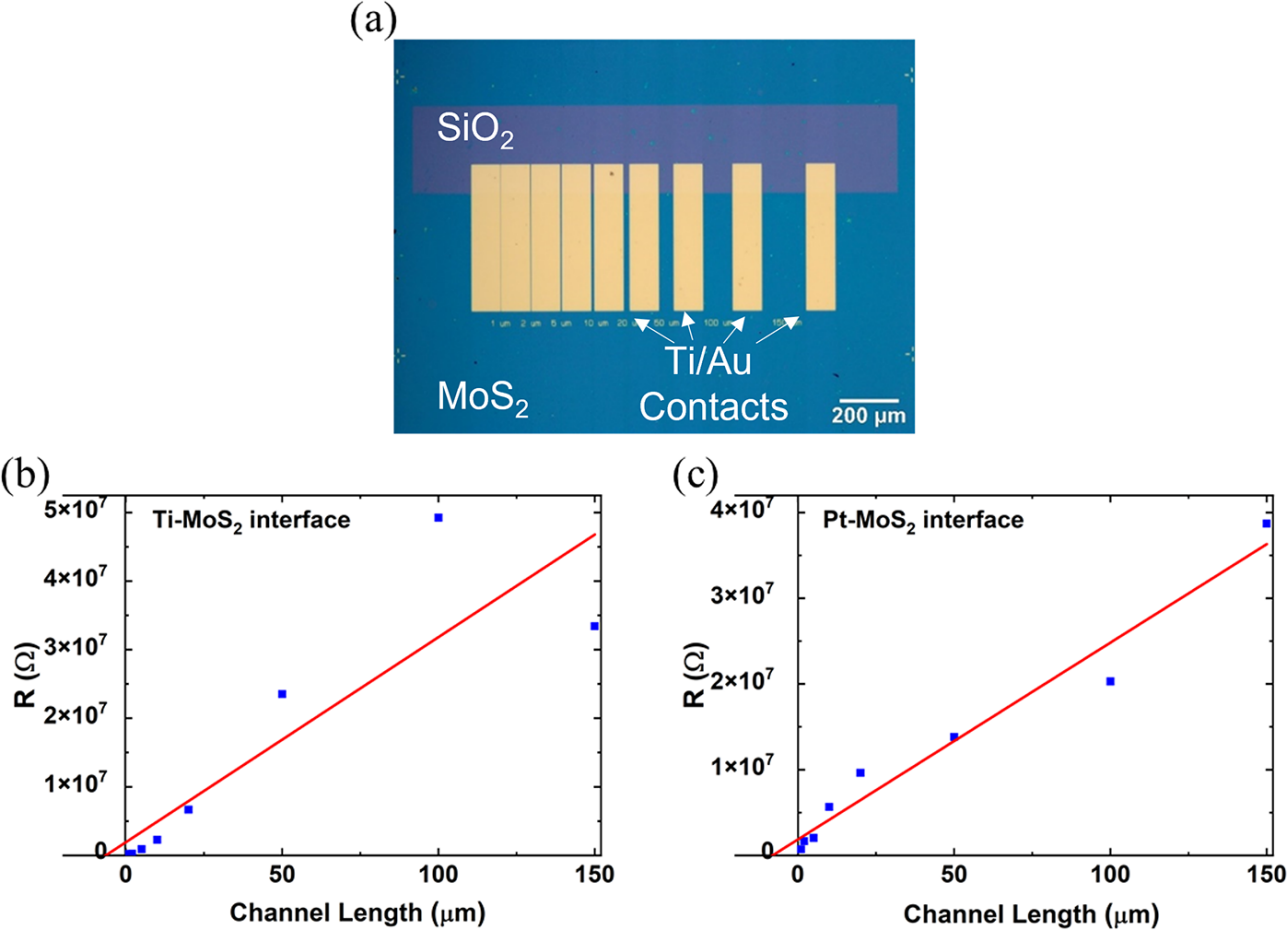
(a) A TLM grid pattern on monolayer MoS_2_ with variable channel lengths from 1 to 150 μm. These structures are used to extract the contact resistivity at the metal–semiconductor interface and the sheet resistivity of the semiconducting material. TLM plots for (b) Ti–MoS_2_ and (c) Pt–MoS_2_ devices. The resistances are plotted for various channel lengths. Contact resistance is extracted from the *y*-axis intercept of the linear fit (divided by two), and the sheet resistance is calculated by taking the slope of the linear fit line and dividing it by the channel width.

To understand limitations in electronic transport, the solar cell is divided into a resistance network consisting of the measurement probes, contacts pads, busbars, grid fingers, contact resistance at the metal–semiconductor interface, and sheet resistance. The total resistance from the probes, contact pads, busbars, and fingers is calculated as 225 Ω, showing that the bulk of the series resistance comes from the contact and sheet resistance. To extract these values, the transfer length method (TLM) is used.^20^Figure 6a shows a TLM grid on monolayer MoS_2_ with variable channel lengths from 1 to 150 μm. Details on the fabrication of the TLM grids are shown in section S6. These measurements are performed with both Ti/Au contacts and Pt contacts under 0.5 V bias in dark, room temperature conditions.

By analysis of the data shown in Figure 6, the average contact resistivity of the Pt–MoS_2_ and Ti–MoS_2_ interface is calculated to be 45.33 and 17.5 Ω·cm^2^, respectively. The average sheet resistance is estimated to be 2.34 × 10^8^ Ω/□. The high sheet resistance in these devices contributes significantly to their poor electronic transport performance. Sheet resistance should be sufficiently low for good lateral transport, preferably in the 50–100 Ω/□ range.^21^ The high contact resistance measured here is not a concern, as these Schottky devices do not use ohmic contacts by design.

**Figure 7:**
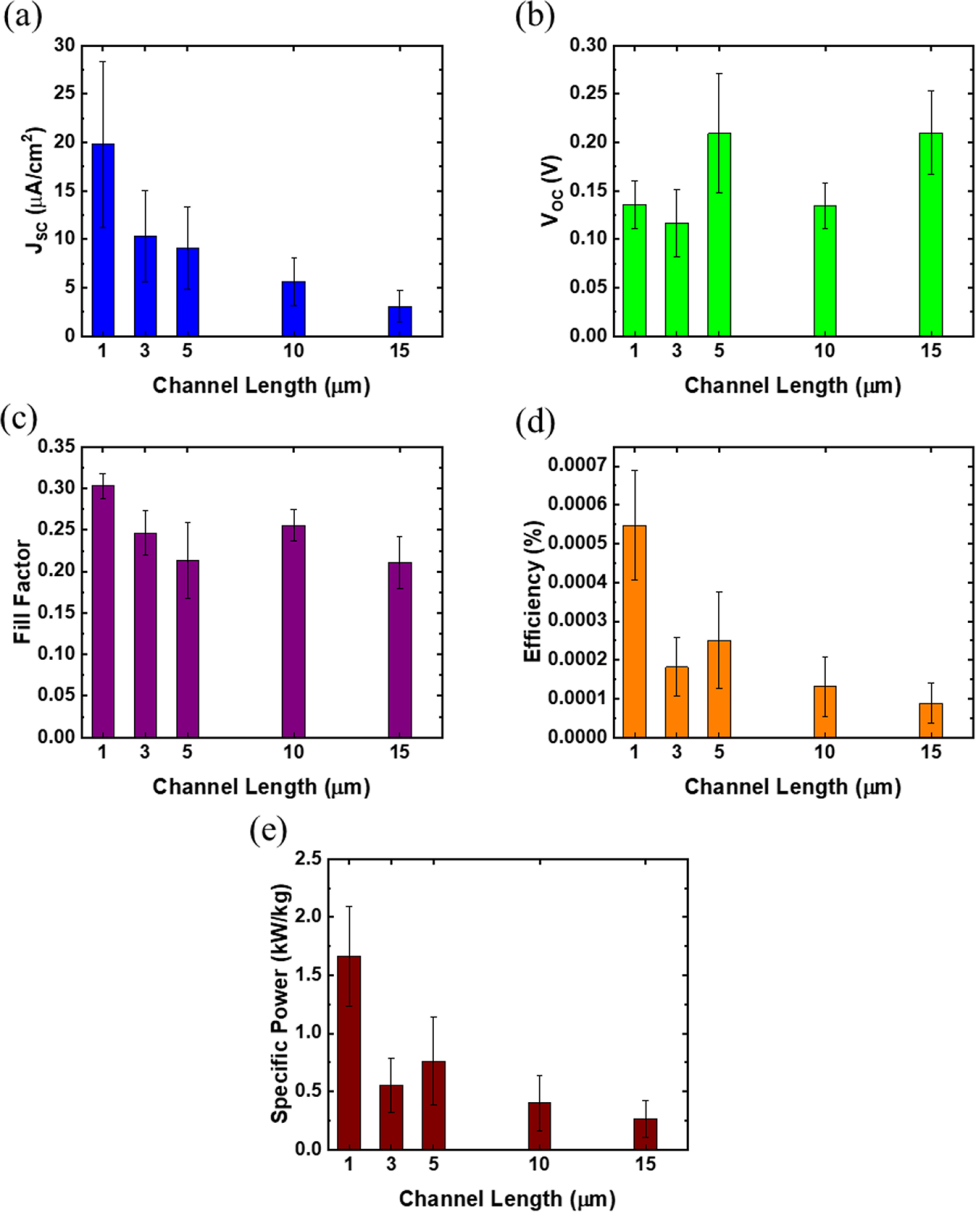
(a) *J*_SC_, (b) *V*_OC_, (c) fill factor, (d) efficiency, and (e) specific power vs channel length for seven devices of each channel length under 1 sun equivalent AM1.5D illumination.

Next, the device performance was investigated with respect to the channel length or the spacing between each Ti and Pt finger. Devices were fabricated with five different channels lengths: 1, 3, 5, 10, and 15 μm. In each case the total active area of the devices was 0.15 mm^2^. Figure 7 plots the mean *V*_OC_, *J*_SC_, fill factor, efficiency, and specific power vs channel length for a total of 35 devices. Data from seven devices were included in each column (channel length) for drawing statistical significance.

The *J*_SC_ has a clear dependence on channel length as can be seen in Figure 7a. As the channel size increases, the devices produce less current under the same illumination conditions and total device active area. This is a result of the short diffusion length of the carriers in the CVD-grown 2D active material. When the channel length becomes smaller than the diffusion length, a significant majority of the generated carriers can be collected by the contacts before they recombine, and the *J*_SC_ should then saturate with decreasing channel length. From the trend in Figure 7, the diffusion length is smaller than 3 μm. Spatial mapping of photocurrent in these devices indicates a lateral diffusion length of about 1.0 μm (see section S7). The *V*_OC_ and fill factor do not have a clear dependence on channel length under the 1 sun illumination conditions. The efficiency and specific power follow the same trend as seen for *J*_SC_.

The *V*_OC_ values reported here are particularly high for CVD-grown monolayer MoS_2_, with a peak measured value of 290 mV in a 5 μm channel length device. Cho et al. fabricated a WSe_2_/MoS_2_ heterojunction with 5 to 10 exfoliated layers of WSe_2_ and MoS_2_ each and achieved a *V*_OC_ of 360 mV under 1-sun illumination without any passivation.^22^ We have not found any report of CVD-grown monolayer 2D active material-based PV to compare our results with and believe our result is the first ever 2D PV device fabricated by using a large-area synthesis technique.

Specific power, or power generated per unit mass, is a critical metric for PV in weight or volume constrained applications, such as space solar power, building-integrated PV, and vehicle-integrated PV. Reese et al. recently compared different existing technologies for high specific power PV.^23^ Nassiri Nazif et al. also recently demonstrated high specific power 2D PV despite low power conversion efficiency and presented a benchmarking study comparing 2D PV with other existing thin-film PV technologies in terms of specific power and efficiency.^7^ The cell active material’s specific power is a significant part of the overall module/package specific power. Compared to the record state-of-the-art GaAs and Si cells’ active material specific powers of 54 and 2.5 kW/kg,^5^ respectively, the yet unoptimized devices presented here have already achieved a specific power of 1.58 kW/kg. We emphasize that only the mass of the MoS_2_ layer and the contact metals are considered here in calculating the specific power of our devices and that of competing materials. Because these devices can be transferred to any arbitrary substrate, we see no barrier to the use of large-area 2D PV with ultralight flexible substrates, such as few micrometers thick polyimide; this is indeed an area of active research in our group and others.^7,24,25^

### 3.2 Model Projection and Analysis

**Figure 8:**
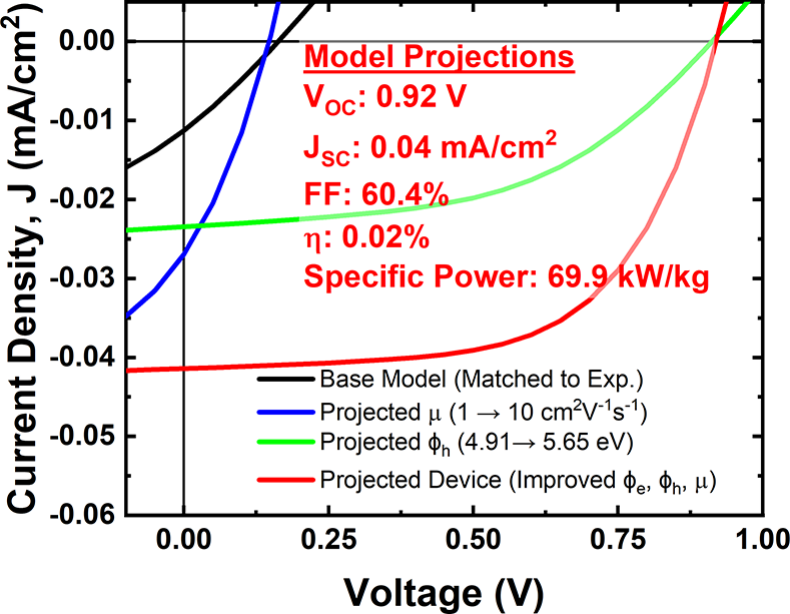
Projected performance of an optimized monolayer MoS_2_-based solar cell with asymmetric Ti–Pt contacts under AM1.5D solar irradiance.

The semiconductor device physics model discussed in section 2 is critical to understanding these 2D PV device results and informs the design of future devices with significantly enhanced performance. Figure 8 shows a modeled *J*–*V* plot of a similar device structure. To match the modeled *J*–*V* relationship to that of the experiment under 1 sun AM1.5D illumination, several parameters are in the model are adjusted. The best fit, as shown in Figure 8, is a result of the following parameters: Φ_e_ as 3.82 eV, Φ_h_ as 4.91 eV, carrier mobility of the MoS_2_ layer as 1 cm^2^ V^–1^ s^–1^ (matching our measured values), and lifetime of 1 μs. These work function values are within the range of measured work function data shown in section S8, signifying lower work function than pristine postsputter metal surfaces but higher work function than that of a sample that has been exposed to ordinary environmental conditions for an extended period. The lower work function of the Pt contact results in a significant degradation of *V*_oc_ in these devices, as shown in Figure 4b. The best-fit mobility and lifetime results in a diffusion length of 1 μm, which matches the higher *J*_SC_ shown in Figure 7 for 1 μm channel lengths and our device photocurrent map, as shown in section S7.

From the experiment-matched model, we systematically sweep parameters to optimize the device performance for a monolayer MoS_2_-based solar cell. The highest impact on the device performance, especially *V*_OC_, comes from increasing the hole collector metal’s work function to that of pristine Pt at 5.7 eV, as shown in Figure 8. Increasing mobility to that of higher performing CVD-grown 2D MoS_2_^26^ is shown to improve the *J*_SC_. Finally, Figure 8 shows the projected *J*–*V* plot for the optimized parameters as follows: Φ_e_ as 4.33 eV, Φ_h_ as 5.65 eV, carrier mobility of 10 cm^2^ V^–1^ s^–1^, and lifetime of 1 μs. The model predicts a *V*_OC_ of 920 mV and a *J*_SC_ of 0.4 mA/cm^2^ with a single 0.65 nm thick active MoS_2_ absorber layer. Although the power conversion efficiency of this projected device is only 0.02%, the specific power of this device is 69.9 kW/kg, higher than that of the record III–V solar cells’ specific power. The *J*_sc_ and efficiency of this device will improve dramatically by stacking layers for enhanced photon absorption.

As has been shown, contact engineering has a major impact on the performance of these Schottky PV devices, and with optimized contacts, the *V*_OC_ and *J*_SC_ of these solar cells can be improved substantially.^27,28^ Knowing the exact work function of a metal is a key design component, as that allows us to model the devices accurately. Our initial experiments with improving work functions of as-evaporated metals show promising results. We measured the work functions of as-deposited Ti and Pt as 3.77 and 4.90 eV, respectively. By sputtering the metal films in vacuum followed by *in situ* work function measurements, the same Ti and Pt films show work function of 4.19 and 5.35 eV, respectively. Further details on the work function measurements and sputtering are shown in section S8 and point to a direction for further work. Future work should also consider the potential impact of Fermi level pinning at the metal–semiconductor interface and its impact on device performance, through model and experiment.

Many other opportunities exist for improvement in 2D PV device design and fabrication.^6^ There are other alternative 2D semiconducting materials with lower bandgap that may be more suitable for PV device design than MoS_2_ (∼1.8 eV), such as MoTe_2_ (∼1.1 eV), WSe_2_ (∼1.4 eV), and so on.^29^ It has been shown that 2D TMDCs can absorb nearly 100% of broadband visible light with sub-15 nm thickness.^30^ The absorption profile can be improved by stacking multiple monolayers on top of each other. Our initial studies show that stacked monolayers of CVD-grown MoS_2_ behave as independent layers with a linear increase in absorption with additional layers, rather than behaving as bilayers or trilayers with reduced absorption per layer. Further work is needed on this as well.

The optical absorption can be further enhanced by applying optical coatings to the front or a back reflector can also be used as the back contact for vertical solar cells. Effective light trapping mechanisms may be integrated into the device structures, including nanostructures that can be utilized for enhanced photon capture.^6^ Dielectric encapsulation as shown by McVay et al.^12^ and passivating surface treatments such as the DCE treatment^31^ can significantly improve the carrier transport and thus the overall performance of a 2D PV device.

While most discussions thus far have revolved around lateral transport 2D PV, vertical Schottky-junction 2D PV could be a suitable alternative to p–n junctions.^7,32^ With vertical Schottky junctions, the devices would not be affected by low lateral transport and high sheet resistance. Instead, the generated carriers will have to diffuse through nanometer thick films, unlocking the 2D films’ true potential.

Finally, p–n homojunction/heterojunction 2D PV also holds significant potential.^9^ If the TMDCs can be effectively doped to be both n-type and p-type, a homojunction PV device can be fabricated. Another path forward is to make heterojunction devices, such as WSe_2_/MoS_2_ or WSe_2_/MoSe_2_ heterojunctions. There has been some work on that end but with minimal success.^33,34^

Lastly, all of the approaches mentioned above still hold the potential for being transferred on to transparent and flexible substrates. 2D materials that are only angstroms thick and absorb 5–20% of the incoming light can revolutionize the way PV are made today. Such semitransparent and/or flexible devices can have widespread applications where optical throughput or high bend radius is required. Also, as demonstrated by the potential for high specific power, 2D PV that are synthesized on or transferred to ultrathin support films, such as those used in solar sails,^25^ are an excellent candidate for any weight or space constrained PV applications, especially those in space.

### 3.3 Large-Area Devices

**Figure 9:**
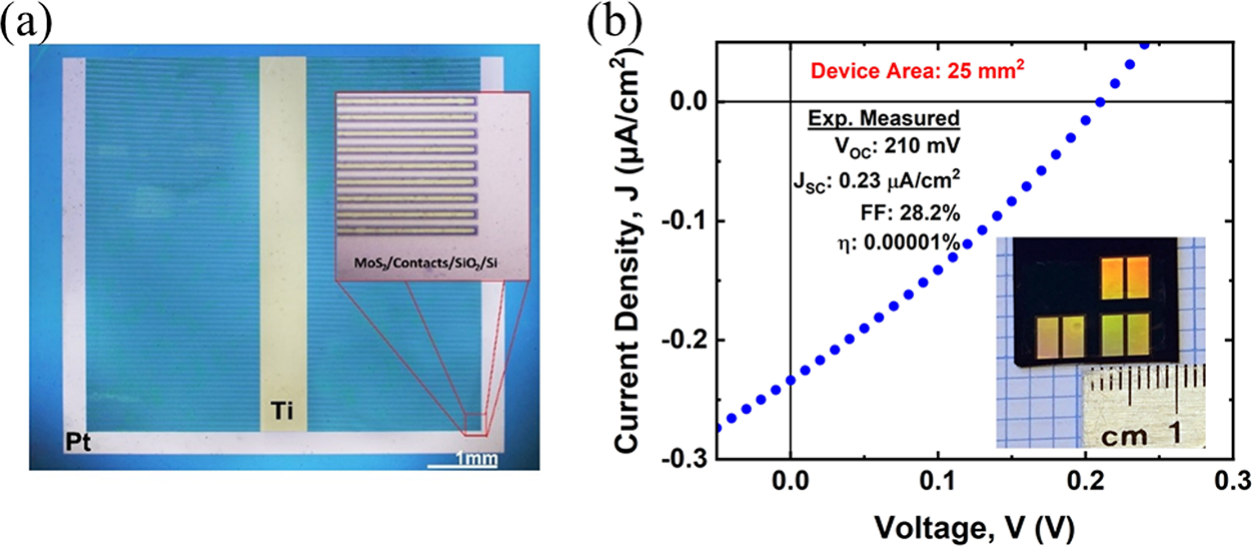
(a) Optical micrograph of a large-area (25 mm^2^) PV device with monolayer MoS_2_ (0.65 nm thick). (b) *J*–*V* performance of this large-area Schottky-junction solar cell under 1 sun equivalent AM1.5D illumination. The channel length between the asymmetric Ti and Pt contacts is 3 μm. The inset shows a photograph of three 25 mm^2^ devices on a SiO_2_-on-Si substrate with a ruler for scale.

To further demonstrate the scalability of the 2D PV device strategy described in this work, we fabricated PV devices with monolayer MoS_2_ that are 5 mm by 5 mm in size—a standard dimension used for some III–V solar cells such as Spectrolab’s C4MJ cell.^35^ Other than an increase in the size of the device contact patterns the device design, material synthesis, and fabrication are the same as in the previously reported smaller devices. Optical microscope and macroscopic photographs of these large-area devices are shown in Figure 9. One can see that these devices are macroscale on the *x*–*y* plane while their active area is only subnanometers in thickness. The *J*–*V* performance of a typical large-area 2D PV device is shown in Figure 9b. These devices generally show higher *V*_OC_ than the small area devices due in part to better perimeter passivation, but their short-circuit current is still limited by the carrier transport and collection constraints of their lateral transport architecture.

More work is needed to optimize these large-area devices. The contact patterns need to be carefully designed, including the fingers’ pitch and width, to prevent leakage or inactive portions of the device, and to maximize the photogenerated carriers’ collection.

## 4 Conclusion

In summary, this work presents the design, modeling, fabrication, and characterization of CVD-grown monolayer MoS_2_-based lateral Schottky-junction PV devices with asymmetric Ti and Pt contacts. The device performance is analyzed under monochromatic and 1 sun equivalent AM1.5D illumination sources. A typical device achieved a *V*_OC_ of 160 mV and a *J*_SC_ of 0.01 mA/cm^2^ while the best performing devices achieved a *V*_OC_ and *J*_SC_ of 290 mV and 0.02 mA/cm^2^, respectively. To understand the low current in the devices, a resistance model is built, and contact resistances are extracted from a TLM measurement, which show very high sheet resistance. Further analysis of device behavior, including work function measurements, shows opportunities for near-term performance enhancement. A 2D PV optoelectronic model is built and validated by the experimental results; the model is then further expanded to design and project future device performance. By only optimizing the metal contact work functions and the carrier mobility of the 2D MoS_2_ film, the model predicts a 0.65 nm thick solar cell active material with ∼70 kW/kg specific power, exceeding the 54 kW/kg record specific power of GaAs-based solar cell active material. With further thickness optimization and module encapsulation, ultrahigh-specific-power solar cells can be achieved with these 2D TMDC materials. Finally, to prove the scalability of these devices toward large-area, practical deployment, a 25 mm^2^ active area cell is fabricated and characterized under 1 sun illumination. Initial results show a *V*_OC_ of 210 mV without optimization, which is very promising for future large-area 2D material-based solar cells.

## 5 Methods

### 5.1 Film Synthesis and Transfer

The 2D MoS_2_ films are synthesized in an MTI OTF-1200X-II dual zone split tube furnace with the addition of a low-temperature third zone by wrapping the tube with a Grainger SLR series silicone heating blanket. The ACS reagent, ≥99.5% molybdenum(VI) oxide (MoO_3_), and the 99.98% trace metals basis sulfur (S) powder were purchased from Sigma-Aldrich. More details on the film synthesis are provided in our prior work.^13^ A surface-energy-assisted film transfer process demonstrated by Gurarslan et al.^14^ was used to transfer the monolayer MoS_2_ films from their sapphire growth substrate onto the PV device metal contacts on an SiO_2_-on-Si substrate.

### 5.2 Device Fabrication

The device fabrication is performed in this sequence: (i) substrate cleaning with solvents (acetone and IPA) and O_2_ plasma, (ii) patterning for the Pt contacts using a RAITH VOYAGER 100 electron beam lithography (EBL) tool, (iii) 50 nm Pt deposition using an Angstrom Engineering Nexdep electron beam evaporation (EBE) tool at 0.5 Å/s rate, (iv) second layer alignment EBL for the Ti contacts, (v) 50 nm Ti evaporation using the same EBE tool at 1 Å/s rate, (vi) immediate transfer of the monolayer MoS_2_ film on top of the Pt and Ti contacts, and finally (vii) device annealing at 200 °C for 1 h in ambient room atmosphere.

### 5.3 Device Characterization

The device current–voltage relationships are measured by using a Keithley 2450 sourcemeter. The 1 sun AM1.5D illumination conditions are simulated in a TS-Space Systems Unisim tunable solar simulator with two tunable lamps and three tunable LEDs. All measurements are performed at room temperature in air.

## Supporting Information Available

S1: Film Characteristics; S2: Fabrication Details; S3: All-in-One Optoelectronic Devices; S4: Additional Details on the Optoelectronic Model; S5: Device Performance under 660 nm Monochromatic Excitation; S6: Transfer Length Method (TLM) Grid Fabrication Process; S7: Photocurrent Spatial Map; S8: Work Function Measurements (PDF)
