# Supplementary material for: Large-Area, High-Specific-Power Schottky-Junction Photovoltaics from CVD-Grown Monolayer MoS2
Source: ACS Appl Mater Interfaces. 2022 May 20;14(21):24281–9. doi: 10.1021/acsami.2c01650 (PMC9164198; doi:10.1021/acsami.2c01650)
Supplement: Supplementary file 1 — am2c01650_si_001.pdf [file am2c01650_si_001.pdf]

Supporting Information

Large-area, high-specific-power Schottky-junction photovoltaics from CVD-grown monolayer MoS_2

Kazi M. Islam,[†]* *Timothy Ismael,*[†] *Claire Luthy,*[†] *Orhan Kizilkaya,*[‡] *and Matthew D. Escarra*[†]

[†]Department of Physics and Engineering Physics, Tulane University, New Orleans, LA 70118, USA, and [‡]Center for Advanced Microstructures & Devices, Louisiana State University, Baton Rouge, LA 70806, USA

*kislam@tulane.edu

S1. Monolayer MoS_2 Film Characterization

Photoluminescence and Raman Spectra

Figure S1(a) shows normalized photoluminescence (PL) intensity of a CVD-grown monolayer MoS_2 sample, showing the excitonic A-peak at 672 nm. The PL peak has 21.6 nm full-width at half maximum (FWHM), confirming good optical quality. This PL is a signature characteristic of all of the monolayer TMDC films used in this work.

The Raman spectra in Figure S1(b) shows the E^1_{2G} and A_{1G} peaks, located at 392 & 411 cm^{-1}, respectively. The 19 cm^{-1} spacing between the E^1_{2G} and A_{1G} peaks is consistent amongst the samples used in this work and is consistent with our previous report[1], confirming the monolayer thickness of these films.

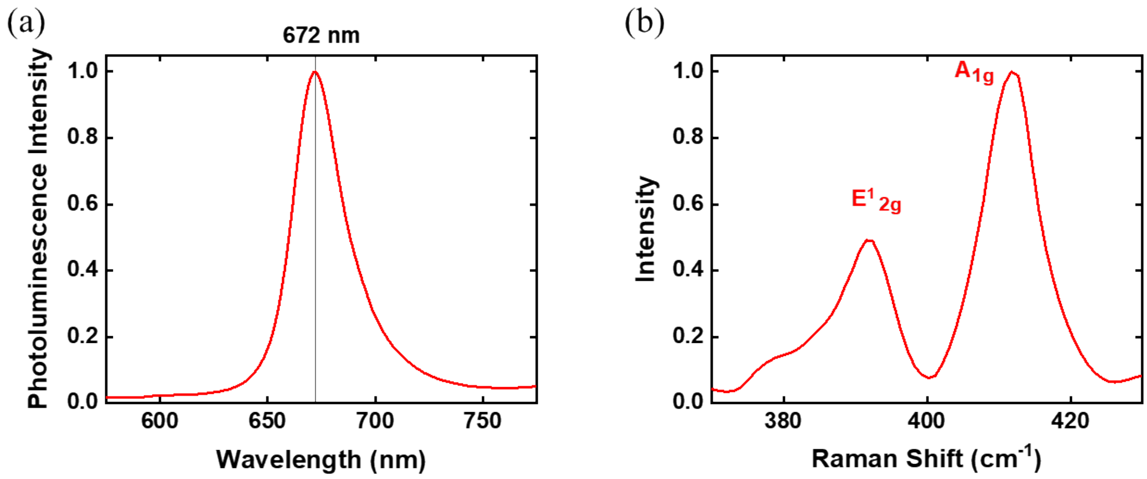

Figure S1: (a) Photoluminescence (PL) spectra of a CVD-grown monolayer MoS_2 sample with an emission peak near 672 nm, using a 405 nm excitation source. (b) Raman characterization of the MoS_2 samples on SiO2-on-Si substrates using a 532 nm laser source.

Carrier Mobility Extraction

The field-effect mobility (μ_{EF}) is an essential metric for quantifying the electronic quality of semiconductor materials, including 2D TMDC materials. Typically, field-effect transistors (FETs) are fabricated to extract the mobility and give insight into the material's electronic transport characteristics. The μ_{EF} can be calculated from the measured transfer characteristics, I_{DS}-V_{GS} sweeps for a given V_{DS}, using the FET model:

$$\mu_{EF} = \frac{dI_{DS}}{dV_{GS}} \frac{L}{W C_{ox} V_{DS}} \quad \text{(S1)}$$

Where L and W are the length and width of the channel, respectively, and C_{ox} is the dielectric capacitance per unit area of the SiO_2 dielectric layer, given by $C_{ox} = \varepsilon_0 \varepsilon_r / d_{ox}$, where ε_0 is the dielectric constant of vacuum, ε_r is the relative dielectric constant of 3.9 for SiO_2, and d_{ox} is the dielectric layer thickness of 300 nm. I_{DS} is the drain-source current, V_{DS} is the voltage between the drain and source terminals, and V_{GS} is the voltage between the gate and source terminals.

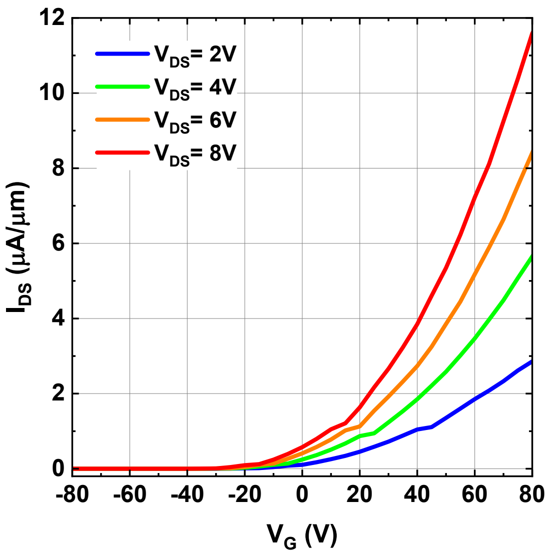

Figure S2: Transfer characteristics of a CVD-grown monolayer MoS_2-based field-effect transistor (FET) with Ti/Au contacts. V_G is swept from -80V to 80V under V_{ds} = 2, 4, 6, and 8 V.

Figure S2 shows a transfer characteristic curve where V_G is swept from -80V to 80V under V_{ds} = 2, 4, 6, and 8 V. With positive gate voltages, the devices turn on, indicating an n-type transistor behavior. From the transfer characteristic curves, the field-effect mobility is extracted as 3 $cm^2V^{-1}s^{-1}$, which is on par for large-area synthesized MoS_2-based transistors.[2]

S2. Fabrication Details

This section contains a step-by-step process flow for fabricating 2D MoS_2-based Schottky-junction photovoltaic devices:

1. A 1 cm x 1 cm SiO_2-on-Si substrate piece is cleaned with solvents (acetone and IPA), and dried with dry N_2, followed by descumming the sample in a plasma asher: 10 sccm O_2, 500 mTorr, 45 W, 60 seconds.
2. The sample is coated with 600 nm PMMA 950 A7. The spin coating recipe is a two-step process:
    a. Step 1: 15 seconds, 500 rpm speed, 100 rpm acceleration
    b. Step 2: 45 seconds, 4000 rpm speed, 500 rpm acceleration
3. The sample is baked at 180 °C for 90 seconds.
4. Next, the device patterns are written using electron beam lithography (EBL). The following process parameters were used in the RAITH VOYAGER 100 EBL tool.
    a. High current (HC) mode (typically 28 nA current), 50 kV beam, 60 µm aperture
    b. Dose: 650 $\mu C/cm^2$; area step size and line spacing: 5-20 nm; Dwell time is calculated by the software using the measured current, set dose, and set area step size/line spacing.
    c. 500 µm write fields are used.
5. Once exposed, the sample is removed from the EBL tool and develop using the MIBK:IPA 1:3 developer for 30 seconds. Rinse IPA on the sample to clean off the residual MIBK.
6. The sample is descummed lightly again before loading inside the electron beam evaporator with the following process recipe: 10 sccm O_2, 500 mTorr, 30 W, 5 seconds

7. 50 nm of Pt is deposited in an electron beam evaporator at 0.5 Å/s deposition rate. The chamber is pumped down to low 1E-6/high 1E-7 Torr base pressure before starting the deposition.
8. Next, excess metal and PMMA is lifted-off in a petri dish with acetone. Usually, pipettes are used for agitation but occasionally this step requires an ultrasonicator to lift off the metals.
9. EBL with 3-point alignment is performed next such that the next metal contacts, Ti in this case, are properly aligned to the already deposited Pt contacts. Steps # 2 through # 6 are repeated with the addition of 3-point alignment EBL.
10. 50 nm of Ti is deposited in an electron beam evaporator at 1 Å/s deposition rate. The chamber is pumped down low 1E-6 Torr base pressure before starting the deposition.
11. Excess metal and PMMA is lifted-off again in a petri dish with acetone.
12. The sample is descummed using the recipe given in step # 1 immediately followed by transfer of monolayer MoS_2 films onto them. The synthesis of monolayer MoS_2 and the transfer process is described elsewhere[1].
13. The devices are finally annealed at 200 °C for 1 hour in air to complete the fabrication process.

Optical micrographs of the various stages of the fabrication process for the asymmetric contact photovoltaic devices:

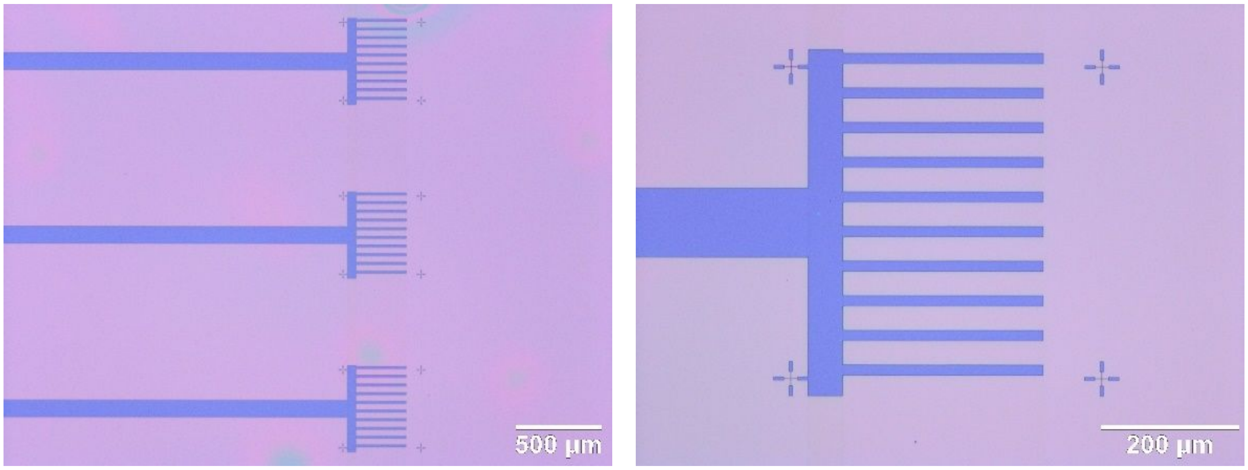

Figure S3: Fingers and busbars of the photovoltaics devices after the 1st EBL step (post-development)

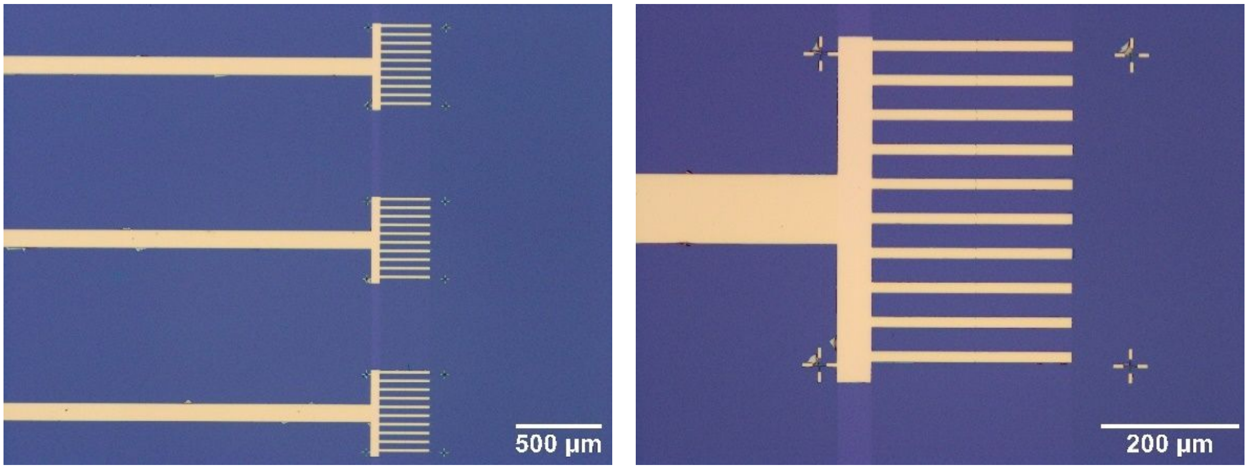

Figure S4: Fingers and busbars of the photovoltaics devices after the 1st metal (Pt) deposition (post liftoff)

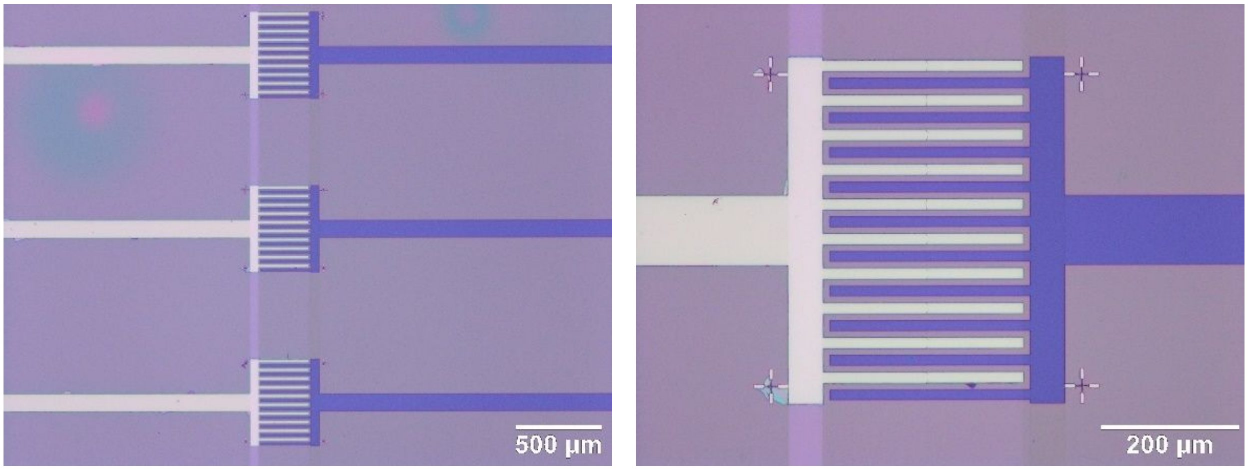

Figure S5: Fingers and busbars of the photovoltaics devices for both contacts after the 2nd EBL step with 3-point alignment (post-development)

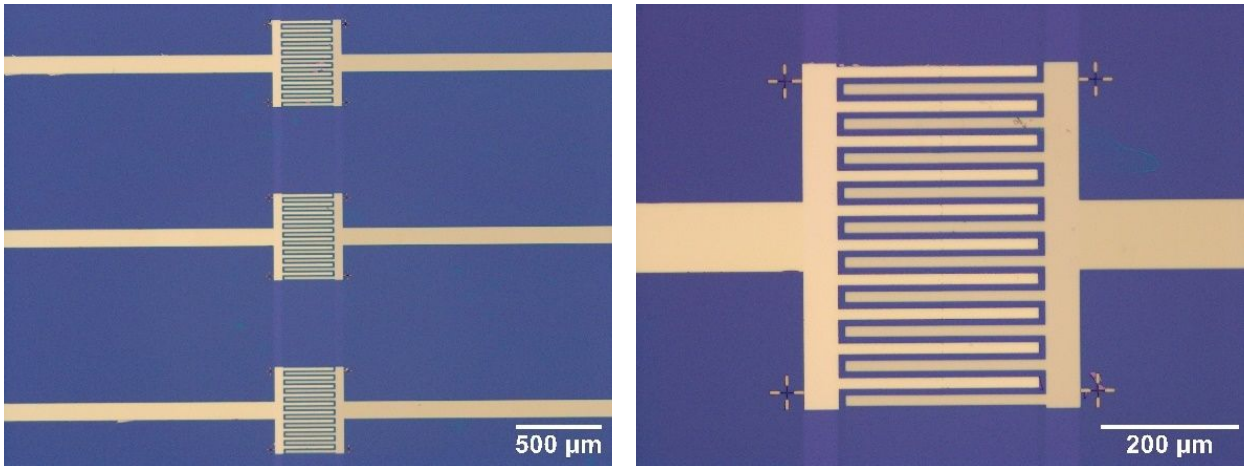

Figure S6: Fingers and busbars of the photovoltaics devices for both contacts after the 2nd metal (Ti) deposition (post liftoff)

S3. All-in-One Optoelectronic Devices

(a) (b)

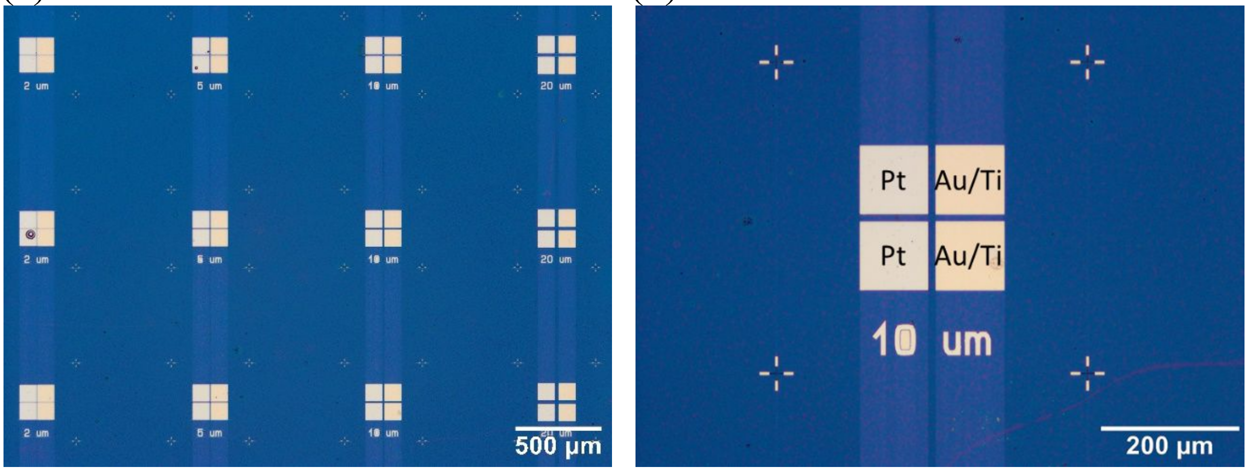

Figure S7: An array of 2D devices (a) and a zoom-in on one device with 10 µm channel length (b) with both symmetric (Ti-Ti and Pt-Pt) and asymmetric (Ti-Pt) contacts.

Figure S7 shows the all-in-one optoelectronic device architecture. By contacting the left two or right two symmetric contact pads (i.e., probing on the pads on the same column), the device can be operated as a transistor (with an external gate contact) or a photodetector, while by contacting the asymmetric contact pads between left and right (i.e., probing on the pads on the same row) the device can be used as a photovoltaic device or a photo-emitter (if the underlying active materials have good radiative efficiency) – all while operating under the right biasing conditions.

Depending on which contacts are probed and how the device is biased, the same device thus can be operated as a transistor, photodetector, photovoltaic, or photo emitter.

The devices in Figure S7 have contacts deposited on top of underlying MoS_2 unlike the devices elsewhere in this paper where the MoS_2 films are transferred on top of the contacts. The Au layer on top of the Ti layer only prevents the Ti layer from oxidizing and is not in direct contact with the MoS_2 layer.

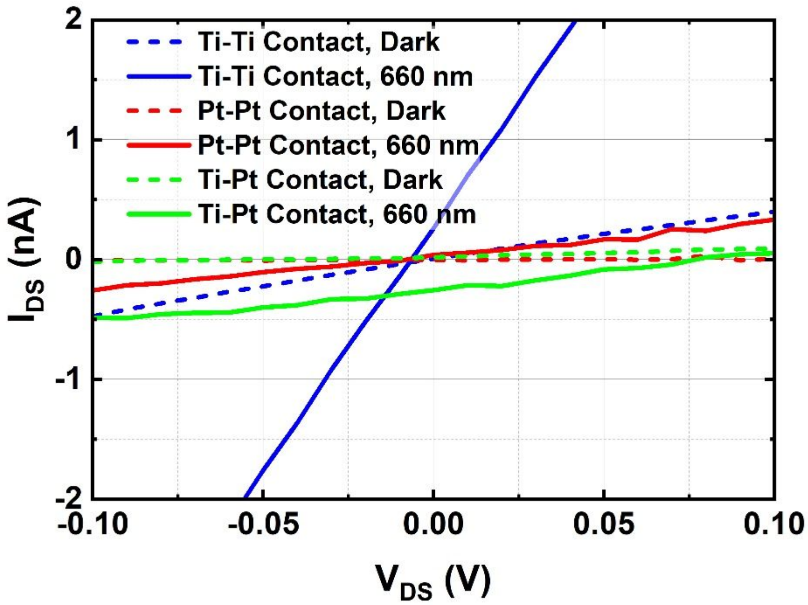

Figure S8: Current-voltage relationship of two symmetric contact devices (Ti-Ti and Pt-Pt) and one asymmetric contact device (Ti-Pt) in dark and under 660 nm monochromatic illumination.

Figure S8 shows an I-V curve of Ti-Ti, Pt-Pt, and Ti-Pt symmetric and asymmetric contact devices in dark and under 660 nm monochromatic illumination. It shows that the symmetric contacts have a photo-induced current as they are more conductive under illumination vs. in the dark, while the asymmetric contact device shows a photovoltage (i.e., an open circuit voltage) unlike the symmetric contact devices.

S4. Additional Details on the Optoelectronic Model

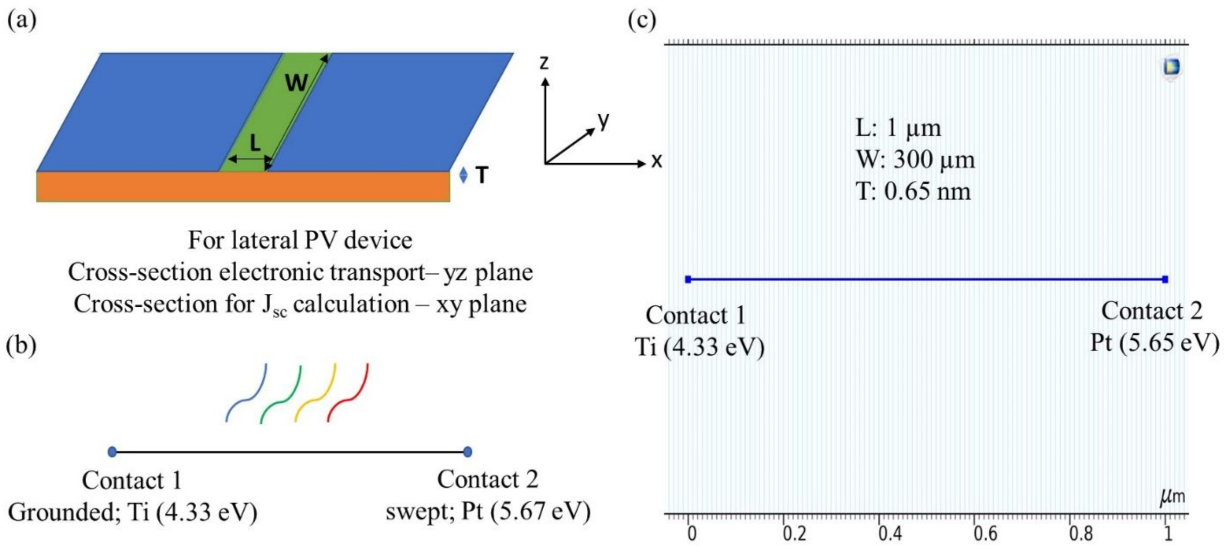

Figure S9: 1D optoelectronic model layout of the Schottky-junction photovoltaic device in COMSOL Multiphysics simulation tool. (a) 3D schematic of the model showing the relevant dimensions, (b) 1D model showing illumination profile, and (c) a screenshot of the 1D model from the COMSOL Semiconductor Module interface.

In the optoelectronic model, the planar photogeneration is assumed to be homogeneous between the asymmetric contacts with a monolayer MoS_2 thickness of 0.65 nm to account for the lateral junction where the solar cell is illuminated from the top. Here, the photogeneration occurs in the z-direction while the electronic transport happens in the x-direction in the 1D lateral PV model. The cross-sectional area considered for the device simulation is the area encompassing the thickness of the material times the width of the channel, 0.65 nm and 300 μm, respectively, as shown in Figure S9. For J_{SC} calculations, a different area is used; the area is given by the length

and width of the channel (in this case, 1 μm and 300 μm, respectively), which describes the sun-facing area of the device.

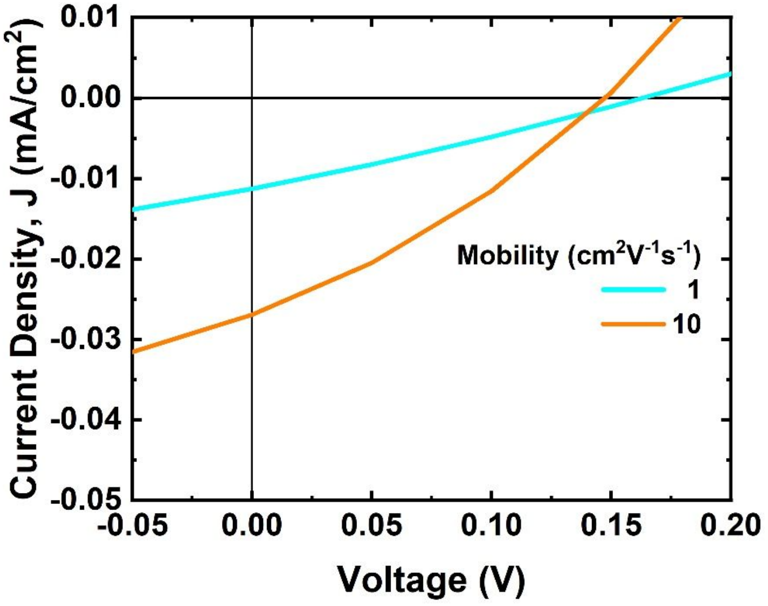

Figure S10: Simulated J-V plots of a Schottky-junction monolayer MoS_2-based solar cell showing the effect of carrier mobility on its performance.

Figure S10 shows the effect of carrier mobility on the current-voltage performance of the Schottky-junction device. The current density-voltage (J-V) plot for 1 $cm^2V^{-1}s^{-1}$ of mobility is a close match with the experimental result. Fig. S8 shows that by only improving the carrier mobility of the 2D material, by a factor of 10, the device current and thereby the efficiency can be improved by a factor of over 2.5x.

S5. Device Performance under 660 nm Monochromatic Excitation

The all-in-one devices' asymmetric contact Schottky-junction configuration are characterized under a monochromatic 660 nm laser light source, as shown in Figure S11(a). The laser power is swept to study the current-voltage relationships at different intensity of the input light.

(a)

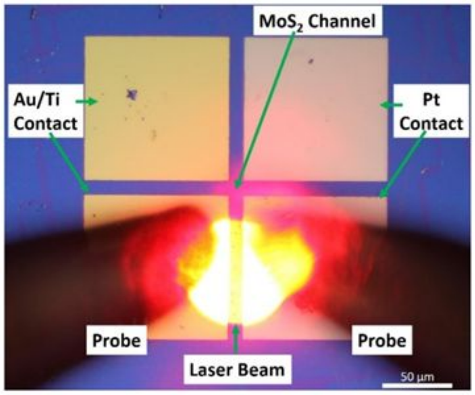

(b)

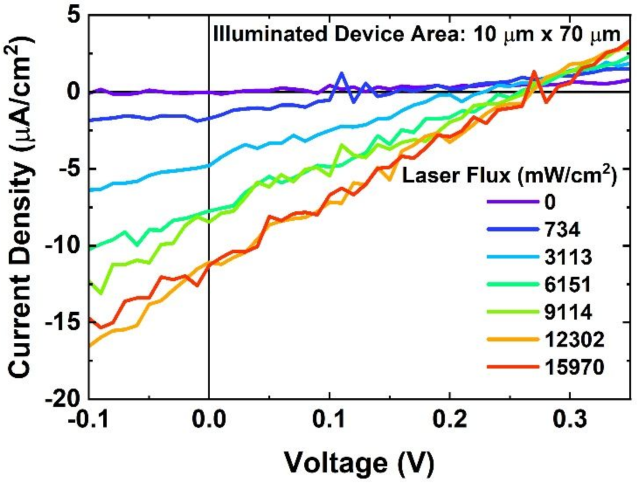

Figure S11: (a) An illuminated asymmetric contact Schottky-junction solar cell with 2D MoS_2. The laser excitation wavelength is 660 nm. (b) J-V plot of the illuminated cell with varying laser excitation up to 15.97 W/cm² flux.

Figure S11(b) shows the J-V plot of the Schottky-junction solar cell that consists of asymmetric Ti and Pt contacts with a single 10 µm x 100 µm channel between them; only 10 µm x 70 µm of the device area is under laser excitation. The contacts are deposited on top of a monolayer film of MoS_2. The monochromatic 660 nm laser excitation was swept up to 15.97 W/cm² intensity on the device. The solar cell shows a V_{OC} of 160 mV and a J_{SC} of 2 µA/cm² at 0.73 W/cm² illumination. At 15.97 W/cm² flux, the V_{OC} and J_{SC} are recorded as 270 mV and 11.5 µA/cm², respectively.

S6. Transfer Length Method (TLM) Grid Fabrication Process

Step 1: Patterning via electron beam lithography (EBL).

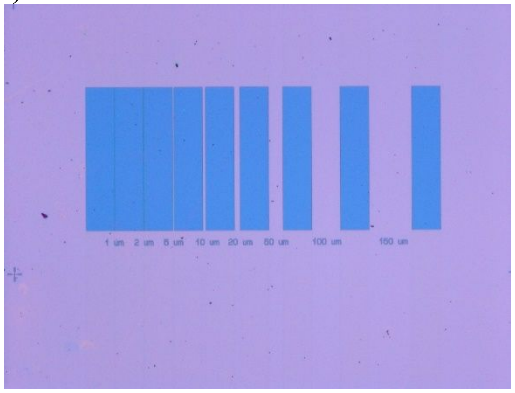

Step 2: Metal contacts are deposited and monolayer MoS_2 is transferred on top.

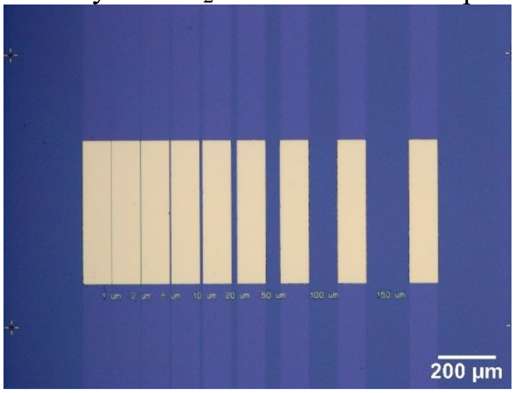

Step 3: 2nd step of EBL is carried out with 3-point alignment to expose and isolate area for MoS_2 etching. This is required for probing the contacts.

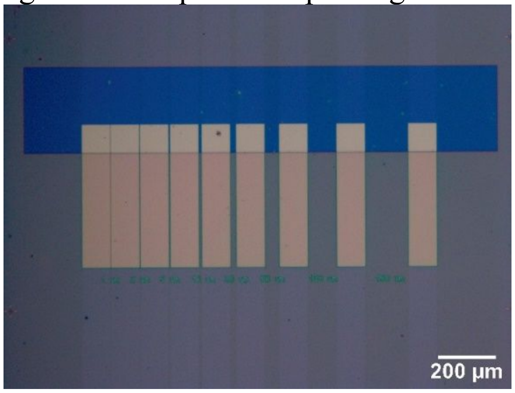

Step 4: MoS_2 is etched from the exposed area using O_2 plasma etching (10 sccm O_2, 500 mTorr, 45 W, 120 seconds).

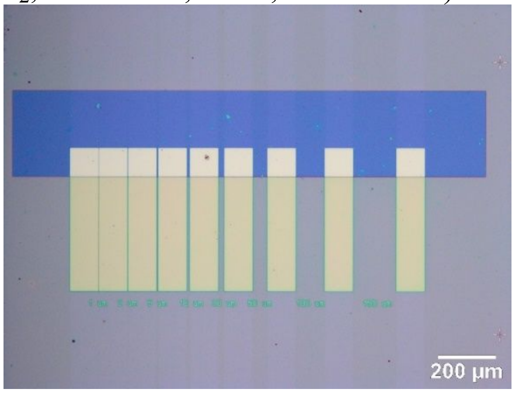

Step 5: PMMA is removed using acetone and toluene.

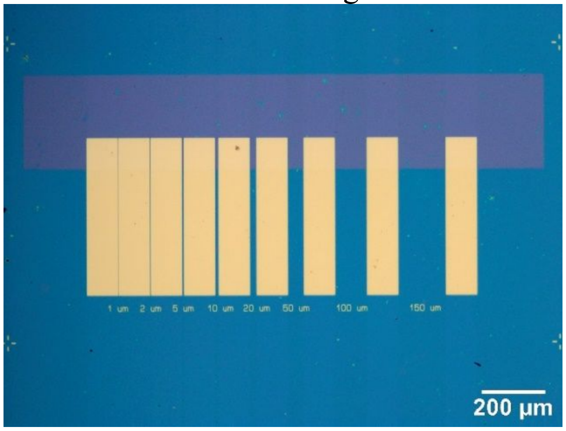

Figure S12: Optical micrographs of the key steps in the TLM pad fabrication process.

S7. Photocurrent Spatial Map

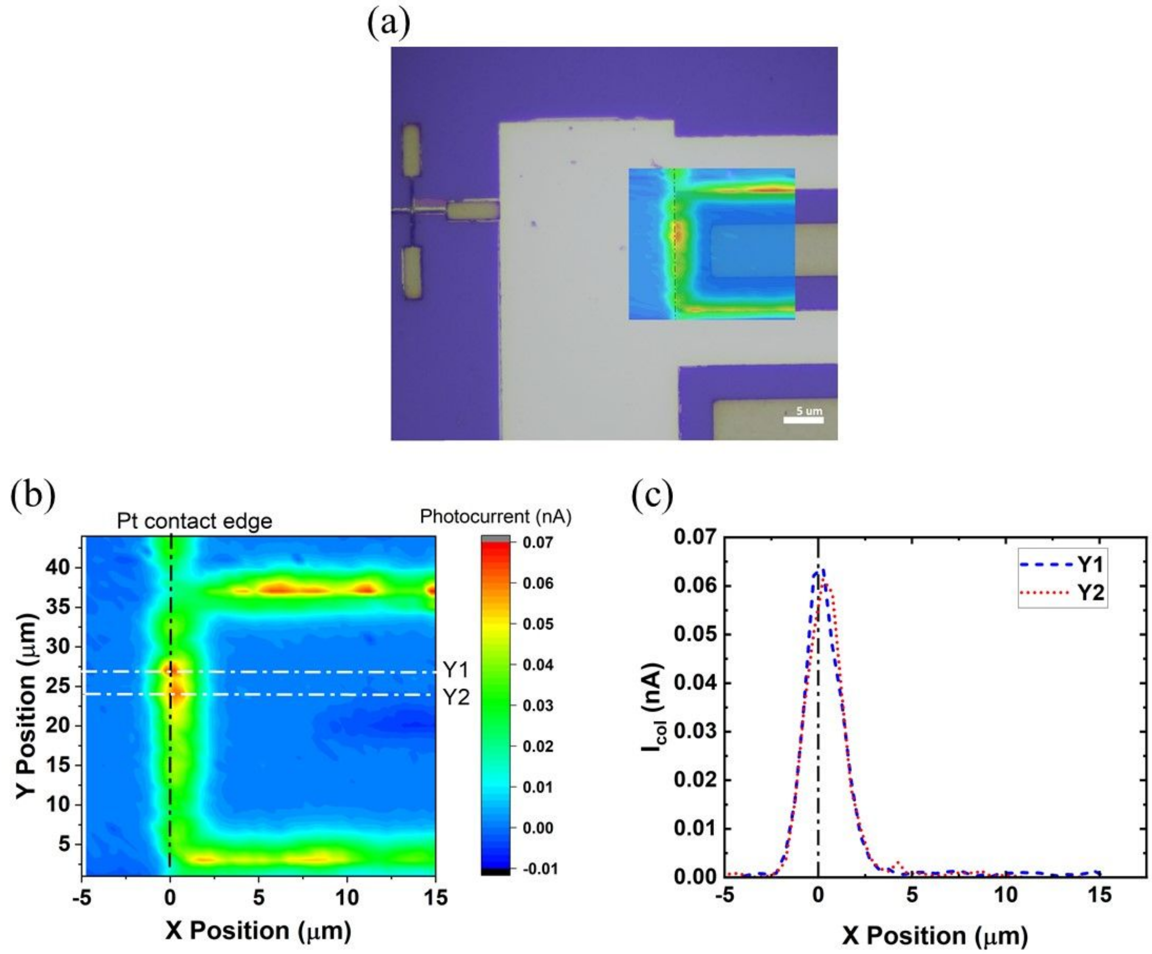

Figure S13: (a) Optical micrograph of a PV device with 2D photocurrent map overlaid. (b) Photocurrent map measured on a 2D PV device with the black dashed line showing the Pt contact edge and the white dashed lines showing where photocurrent is plotted in (c). (c) Photocurrent line profiles as a function of the laser position x, scanned across the Pt contact edge.

Scanning photocurrent microscopy was used to estimate the diffusion length directly by mapping the laser bias induced current (LBIC)[3–5]. The photocurrent was strongest when the laser spot illuminated the MoS_2 layer near the Pt contact edge, and it decreased exponentially as the spot moved away from the contact edge. This resulted in the collected current line profile of the photocurrent as a function of distance from the Pt contact edge as shown in Figure S13(c). The collected photocurrent I_{col} follows the 1D exponential function for $x > 0$, as follows:

$$I_{col} = \frac{eG}{2} exp\,(-x/L_n) \tag{S2}$$

where e is the electron charge and G is the electron generation rate. $L_n = (D_n\tau_n)^{1/2}$ is the diffusion length where D_n is the electron diffusion coefficient and τ_n is the electron lifetime. By fitting the photocurrent curve with a simple exponential function, we extract an L_n of 1.0 μm. The depletion width at the contacts is calculated to be <20 nm in these devices, as depicted in Fig. 3, which is significantly smaller than this measured diffusion length. This ensures that diffusion, and not drift, is the primary means of carrier transport to the contacts in these devices.

S8. Work Function Measurements

The work functions of the metals used in the study are measured via ultraviolet photoelectron spectroscopy (UPS), and findings show that in-situ sputtering of the metals can significantly increase their work function.

The measurements were conducted at the 5-meter toroidal grating monochromator (5m-TGM) beamline at the Center for Advanced Microstructures and Devices (CAMD) at Louisiana State University. The instrument is equipped with a photoemission end station utilizing an Omicron EA125 hemispherical electron energy analyzer with a five-channel detector. The spectra were collected with a constant pass energy of 5 eV and a photon energy of 65 eV. The chamber is kept at a base pressure of 10^{-10} Torr and the surface of the samples was cleaned with ion sputtering (1 keV, Ne^{+}, 10^{-5} Torr) for 3-15 min before the work function measurements. The work function values were determined from the secondary electron cut-off of the UPS spectra of the samples after applying a −10 V bias to the samples to clear the analyzer work function.

The following plots show the before and after sputtering valence band intensity followed by their work functions for three metals of interest for this study: Ti, Pt, and Pd.

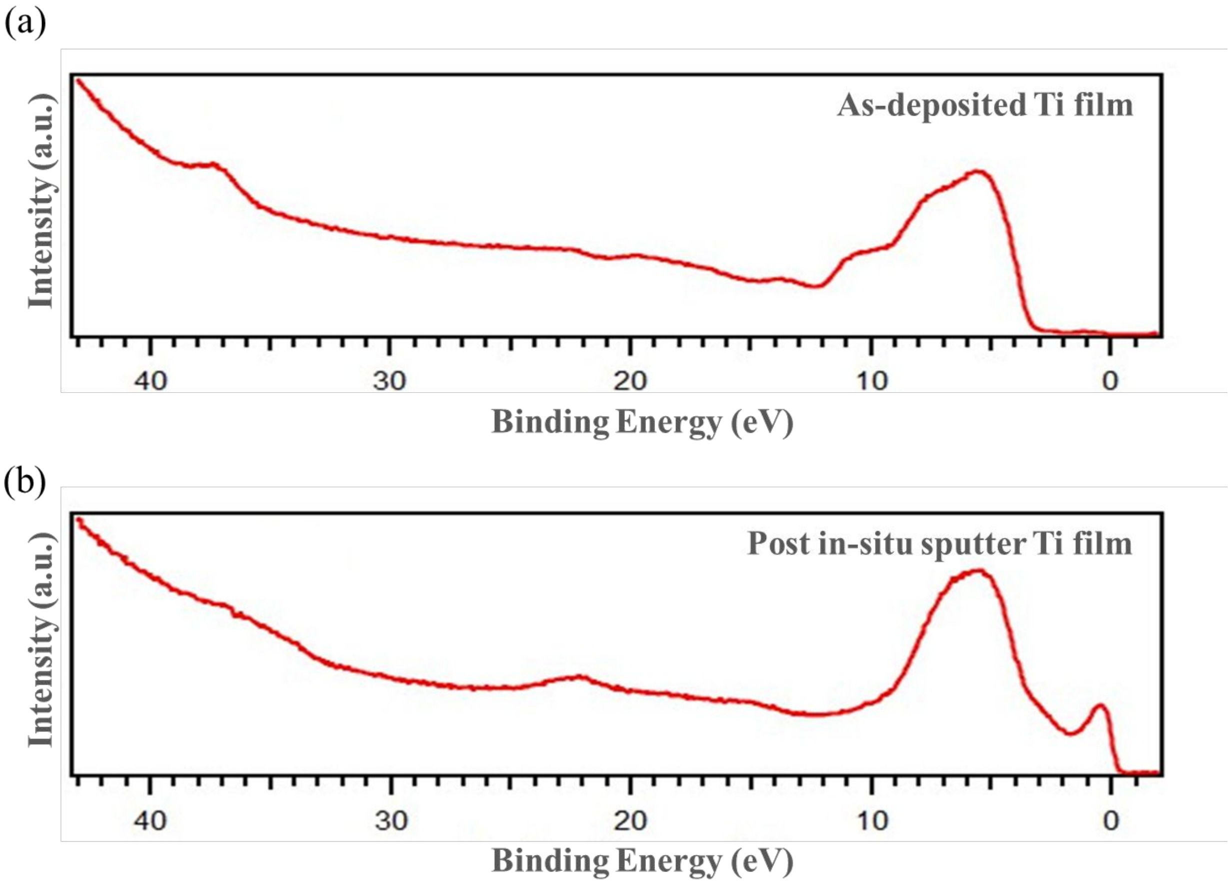

Figure S14: Ti film UPS valence band spectra before (a) and after (b) in-situ sputtering.

(a)

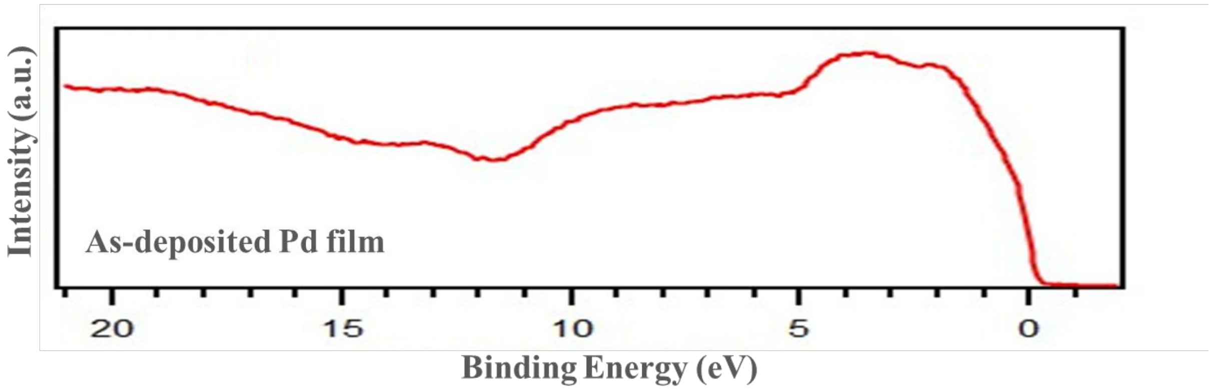

(b)

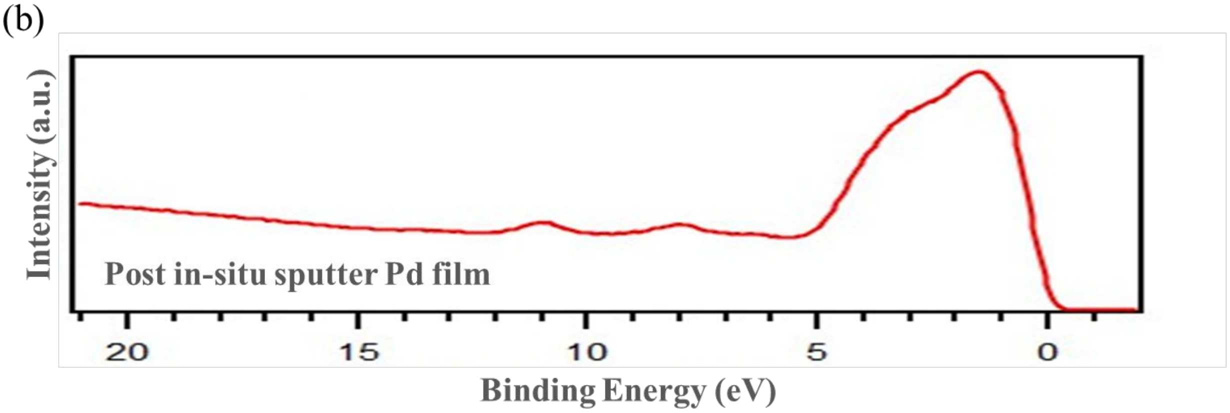

Figure S15: Pd film UPS valence band spectra before (a) and after (b) in-situ sputtering.

(a)

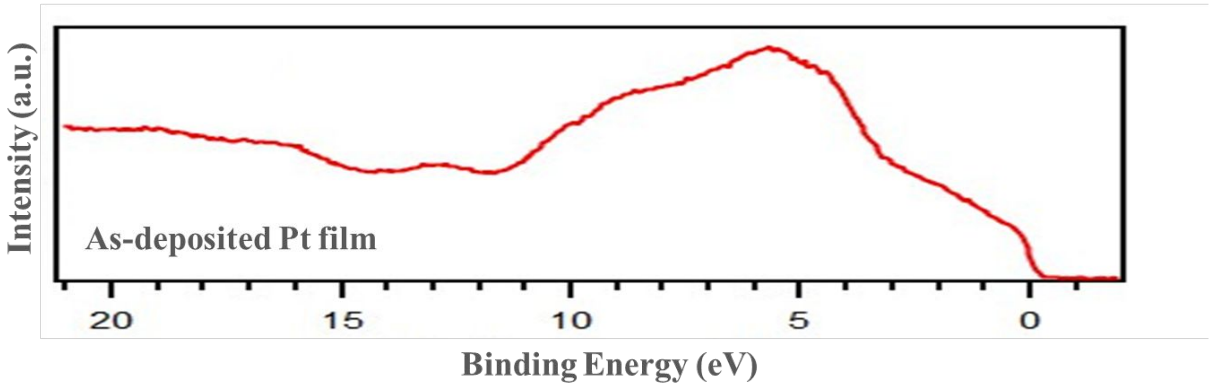

(b)

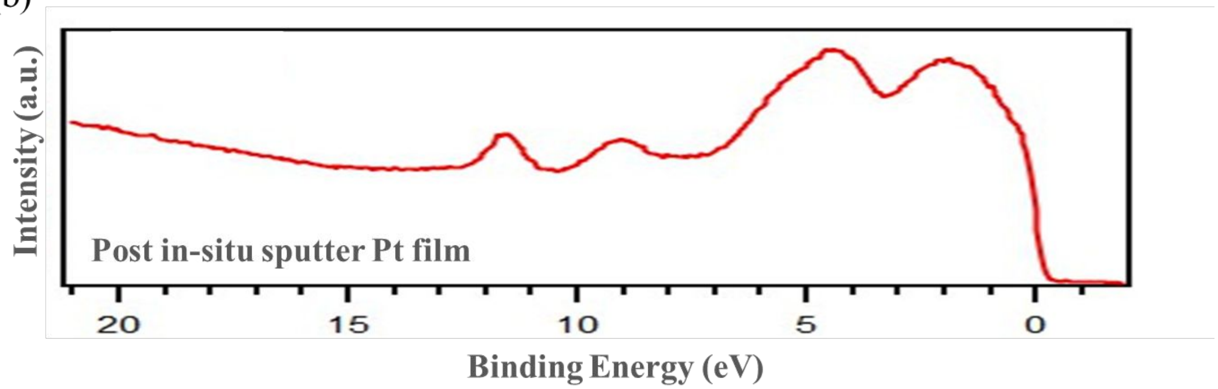

Figure S16: Pt film UPS valence band spectra before (a) and after (b) in-situ sputtering.

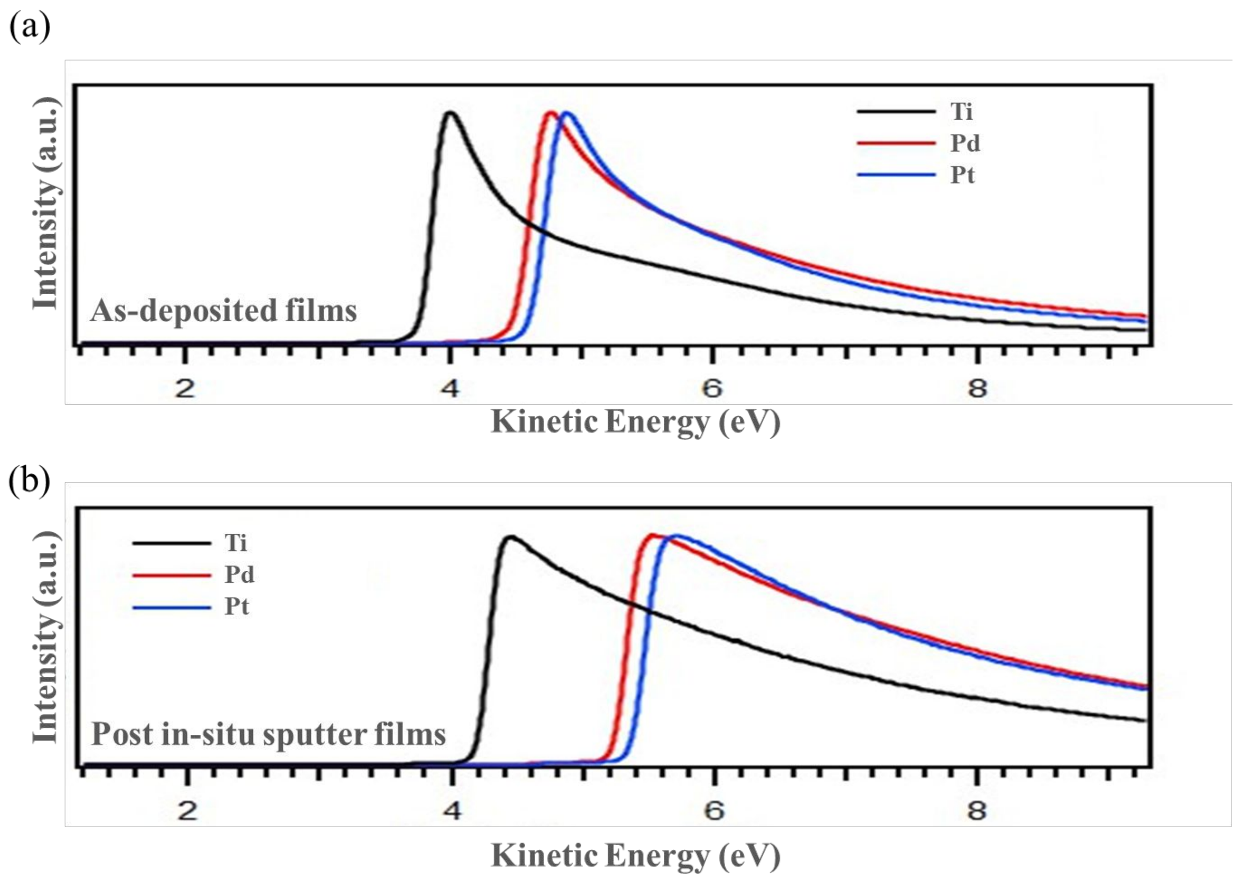

Figure S17: Work function spectra of the metal contact films before (a) and after (b) in-situ sputtering, as measured by UPS.

Table S1: Measured work function of Ti, Pd, and Pt films before and after in-situ sputtering

**Metal/Work Function**	Before in-situ sputter (eV)	After in-situ sputter (eV)
Ti	3.77	4.19
Pd	4.46	5.23
Pt	4.61	5.35

REFERENCES

(1) Islam, K. M.; Synowicki, R.; Ismael, T.; Oguntoye, I.; Grinalds, N.; Escarra, M. D. In-Plane and Out-of-Plane Optical Properties of Monolayer, Few-Layer, and Thin-Film MoS_2 from 190 to 1700 Nm and Their Application in Photonic Device Design. *Adv Photo Res* **2021**, *2* (5), 2000180. https://doi.org/10.1002/adpr.202000180.

(2) Li, N.; Wang, Q.; Shen, C.; Wei, Z.; Yu, H.; Zhao, J.; Lu, X.; Wang, G.; He, C.; Xie, L.; Zhu, J.; Du, L.; Yang, R.; Shi, D.; Zhang, G. Large-Scale Flexible and Transparent Electronics Based on Monolayer Molybdenum Disulfide Field-Effect Transistors. *Nat Electron* **2020**, *3* (11), 711–717. https://doi.org/10.1038/s41928-020-00475-8.

(3) Navas, J.; Guillén, E.; Alcántara, R.; Fernández-Lorenzo, C.; Martín-Calleja, J.; Oskam, G.; Idígoras, J.; Berger, T.; Anta, J. A. Direct Estimation of the Electron Diffusion Length in Dye-Sensitized Solar Cells. *J. Phys. Chem. Lett.* **2011**, *2* (9), 1045–1050. https://doi.org/10.1021/jz200340h.

(4) Gonzalez-Vazquez, J. P.; Anta, J. A.; Bisquert, J. Determination of the Electron Diffusion Length in Dye-Sensitized Solar Cells by Random Walk Simulation: Compensation Effects and Voltage Dependence. *J. Phys. Chem. C* **2010**, *114* (18), 8552–8558. https://doi.org/10.1021/jp100874e.

(5) Park, J.-K.; Kang, J.-C.; Kim, S. Y.; Son, B. H.; Park, J.-Y.; Lee, S.; Ahn, Y. H. Diffusion Length in Nanoporous Photoelectrodes of Dye-Sensitized Solar Cells under Operating Conditions Measured by Photocurrent Microscopy. *J. Phys. Chem. Lett.* **2012**, *3* (23), 3632–3638. https://doi.org/10.1021/jz301751j.
